# PGPR *Kosakonia Radicincitans* KR-17 Increases the Salt Tolerance of Radish by Regulating Ion-Homeostasis, Photosynthetic Molecules, Redox Potential, and Stressor Metabolites

**DOI:** 10.3389/fpls.2022.919696

**Published:** 2022-08-01

**Authors:** Mohammad Shahid, Fatimah S. Al-Khattaf, Mohammad Danish, Mohammad Tarique Zeyad, Ashraf Atef Hatamleh, Abdullah Mohamed, Sajad Ali

**Affiliations:** ^1^Department of Agricultural Microbiology, Faculty of Agricultural Sciences, Aligarh Muslim University, Aligarh, India; ^2^Department of Botany and Microbiology, College of Sciences, King Saud University, Riyadh, Saudi Arabia; ^3^Section of Plant Pathology and Nematology, Department of Botany, Aligarh Muslim University, Aligarh, India; ^4^ICAR-NBAIM, Mau, India; ^5^Research Centre, Future University in Egypt, New Cairo, Egypt; ^6^Department of Biotechnology, Yeungnam University, Gyeongsan, South Korea

**Keywords:** halotolerant PGPR, bioactive molecules, colonization and biofilm formation, phenolics and alkaloids, stressor metabolites

## Abstract

Among abiotic stresses, salinity is a significant limiting factor affecting agricultural productivity, survival, and production, resulting in significant economic losses. Considering the salinity problem, the goal of this study was to identify a halotolerant beneficial soil bacterium to circumvent salinity-induced phytotoxicity. Here, strain KR-17 (having an irregular margin; a mucoid colony; Gm-ve short rod; optimum temperature, 30°C; pH 7.0; no any pigmentation; showed a positive response to citrate utilization, catalase, starch, sucrose, lactose, and dextrose, etc.) recovered from rhizosphere soils of the potato-cultivating field, tolerated surprisingly a high (18% NaCl; 3.-M concentration) level of salt and identified as *Kosakonia radicincitans* (Accession No. OM348535). This strain was discovered to be metabolically active, synthesized essential PGP bioactive molecules like indole-3-acetic acid (IAA), siderophore (iron-chelating compounds), ACC deaminase, and ammonia, the quantity of which, however, increased with increasing NaCl concentrations. Here, *Raphanus sativus* L. (radish) was taken as a model crop to evaluate the adverse impact of NaCl, as well as salinity alleviation by halotolerant *K. radicincitans*. Salinity-induced toxicity to *R. sativus* was increased in a dose-dependent way, as observed both *in vitro* and *in vivo* conditions. Maximum NaCl levels (15%) demonstrated more extreme harm and considerably reduced the plant's biological features. However, membrane damage, relative leaf water content (RLWC), stressor metabolites, and antioxidant enzymes were increased as NaCl concentration increased. In contrast, halotolerant *K. radicincitans* KR-17 relieved salinity stress and enhanced the overall performance of *R. sativus* (L.) by increasing germination efficiency, dry biomass, and leaf pigments even in salt-challenged conditions. Additionally, KR-17 inoculation significantly (*p* ≤ 0.05) improved plant mineral nutrients (Na, K, Ca, Mg, Zn, Fe, Cu, P, and N). Following inoculation, strain KR-17 enhanced the protein, carbohydrates, root pigments, amino acids (AsA and Lys), lipids, and root alkaloids in *R. sativus* (L.). Besides these, due to PGPR seed priming in NaCl-stressed/non-stressed conditions, membrane damage, RLWC, stressor metabolites, and antioxidant defense enzymes were dramatically reduced. The strong biofilm-forming capacity of *K. radicincitans* could result in both *in vitro* and *in vivo* colonization under NaCl stress. Conclusively, halotolerant *K. radicincitans* KR-17 may probably be investigated affordably as the greatest way to increase the production of radish under salinity-stressed soils.

## Introduction

In recent times, the rising global population has been accompanied by increased food consumption, and Oleraceous plants are vital to satisfy this growing need. *Raphanus sativus* L. (radish) is a significantly important root, leafy, and fruit vegetable cultivated in India and South East Asia, belonging to the Brassicaceae family (Lee et al., 2021). Due to its pungent flavor and crisping texture, radish roots are often consumed raw in the form of salad and pickles. Being a leafy vegetable, radish contains a vast variety of phytochemicals like vitamin “A” (Gamba et al., 2021) vitamin “C”, minerals like sulfur (Jaafar et al., 2020), and anthocyanins and glucosinolates and other micronutrients that help enhancing the health of humans. This crop has anti-tumorigenic, antioxidant- and microbiome-regulating, and therapeutic properties, and, due to this very reason, radish is a well-studied crop.

Salinity is an important abiotic factor that reduces the yield and physiological processes of several plants worldwide (Ghosh et al., 2016). According to a recent analysis, salt affects 11.73 million km^2^ of agricultural soil (Hassani et al., 2020). It affects over 20% of the world's agricultural land and about half of the world's irrigated areas. The negative impact of NaCl on plants is the result of two factors: (i) water deficit caused by excessive solute concentrations in the soil and (ii), particularly, Cl and Na+ stress. Furthermore, there are two primary components of salinity stress; (i) ionic, which is connected to the toxicity of the ions produced by salts, and (ii) the osmotic component, in which a high salt concentration in soil solution limits water availability to plants (Miranda et al., 2017). Under a saline environment, high osmotic stress and sodium (Na) toxicity pose a negative impact on crops (Ali et al., 2021). As a result, plants experience a wide range of physiological and biochemical alterations (Chang et al., 2019) in nutrient absorption (Petretto et al., 2019), mobilization, osmotic balance, membrane integrity, oxidative stress, photosynthetic rate (Ji et al., 2018), photorespiration, transpiration (Jan et al., 2020), protein and glucose synthesis, disruption in amino and nucleic acid metabolism and overall growth and, ultimately, reducing the crop output and land-use sustainability.

New and more effective techniques to boost crop yield in salt-stressed soil are critical to long-term agricultural production and food security, especially in saline soils. In numerous crops, plant breeders have attempted to generate salt-tolerant varieties. However, accessibility to salt-tolerant varieties is very limited. Several approaches have been used to promote salt tolerance, and one of them is the use of NaCl-tolerant (halotolerant) compatible microbial inoculants. The evolution of ways and tactics to reduce the detrimental effects of salt stress on plants has thus garnered great focus. Several approaches have been used to promote salt tolerance, and one of them is the use of salinity-alleviating microbial inoculants.

Inoculation of plant growth-promoting (PGP) rhizobacteria plays a significant role in crop development, nutritional management (Bechtaoui et al., 2020), and disease control (Zaidi et al., 2016, 2017). These PGP bacteria infiltrate exo-rhizospheres/endo-rhizospheres of plants and increase crop productivity through a variety of direct/indirect mechanisms. Furthermore, the importance of PGP bacteria in the management of biotic and abiotic stresses is expanding. In such circumstances, the most appropriate approach is to utilize salt-tolerant bacterial (halotolerant) inoculants, which may be beneficial in creating ways to aid plant development in saline soils. Halotolerant PGP bacteria may also relieve the damaging effects of salinity by synthesizing multiple growth-regulating substances (Camaille et al., 2021). The mechanism of NaCl tolerance and its mitigation by rhizobacteria for plants could be the result of a combination of actions, including (i) synthesis of plant hormones (indole-3-acetic acid, abscisic acid, gibberellic acid, and cytokinins), (ii) synthesis of ACC deaminase, which decreases the ethylene level in root tissues, (iii) release of extra polymeric substances (EPS), and (iv) induced systemic resistance (ISR) to fungal diseases by bacterial compounds. The possible mechanism behind salinity alleviation by soil microbes is that halotolerant PGP bacteria express the antioxidant genes that play a significant role in the maintenance of reactive oxygen species (ROS) levels in plants exposed to high salinity stress, thus confirming the significance of NaCl tolerant microbes in free radical scavenging in saline conditions. Furthermore, salinity mitigation can be explained by the fact that enzymes are involved in the neutralization of free radicals, so the amount of free enzymes is reduced, resulting in a decrease in enzyme production, as well as having plant growth-promoting function. Another reason for PGPR-mediated growth promotion is that halotolerant PGP bacteria aid plants under salt stress by breaking down elevated ethylene hormone levels using ACC deaminase, as ethylene inhibits plant growth in stressful situations.

Multiple studies have found that rhizosphere bacteria play a key role in reducing salt stress in a variety of crop plants. For instance, halotolerant PGPR strains (*Kocuriaerythromyxa* and *Staphylococcuskloosii*), following their application to salinity-stressed radish plants, caused improved accumulation of Na^+^, reduced the membrane damage, and increased plant production (Yildirim et al., 2008). Additionally, salt-tolerant *Bacillus oryzicola* strain YC007 augmented the length, biomass, and chlorophyll molecules accumulated in *R. sativus* L. and *Brassica oleracea* L. plants by (i) maintaining intracellular Na^+^ ion concentration and (ii) regulating *SOS1*-dependent salinity-stressed signaling pathways (Baek et al., 2020). In addition, a study conducted by Kaymak et al. (2009) reported that bio-priming of radish seeds with halotolerant PGPRs (*Bacillus subtilis, Burkholderia gladii, Bacillus megaterium, Pseudomonas putida*, and *Agrobacterium rubi*) caused a surprising increase in germination efficiency of plants raised in soils exogenously added with NaCl. Studies on *Triticumestivum* (L.) grown in saline–sodic soil that were fertilized and inoculated with ACC deaminase positive *Bacillus* sp., *Zhihengliuellahalotolerans* and *Staphylococcussuccinus*, either alone or in combination, alleviated the NaCl-induced toxicity, and plants grew and yielded better (Orhan, 2016).

Under saline-induced agricultural practices, the application of salinity-alleviating microbes is a green option for increasing crop productivity; however, additional research into how they act and affect the plant system is needed. Considering the negative impact of salinity-induced stress on agriculturally important crops, especially *R. sativus* (L.) radish, and bearing in mind the importance of halotolerant microbes in salinity alleviation, we isolated NaCl-tolerant *Kosakonia radicincitans*. It is a Gram-ve, rod-shaped plant growth-promoting soil bacterium (residing inside/outside the rhizosphere soil of several important vegetable crops), which belongs to the new genus *Kosakonia* and family Enterobacteriaceae. Numerous *Kosakonia* species have been recovered from different soil sources and are reported to increase the growth of *Vigna radiata* (Shahid et al., 2021a), *Medicago sativa* (Noori et al., 2018), *Solanum lycopersicum* (Berger et al., 2017; Silambarasan et al., 2022), *R. sativus* (Berger et al., 2015), etc., by synthesizing different types of essential PGP metabolites (IAA, ACC deaminase, siderophore, and ammonia, etc.).

The study was further intended for specific objectives: - (i) isolation of PGPR strain and its morpho-biochemical characterization (ii) evaluation of salt-tolerance potential of bacterial strains and molecular identification of selected strain (iii) determination of essential bioactive molecules under NaCl-stressed growth medium (iv) assessment of biofilm formation and associated PGP traits under salt stress (v) evaluation of salt-relieving potential of *K. radicincitans* KR-17 on biological attributes and photosynthetic molecules of NaCl-treated radish raised in pot soils (vi) determination of mineral composition in radish grown in absence/presence of NaCl and halotolerant PGPR inoculums (vii) extraction and determination of total soluble protein, carbohydrates, amino acids, and pigments in tissues of bio-inoculated and NaCl-supplemented radish (viii) assessment of *in vivo* biofilm formation and further colonization.

## Materials and Methods

### *In vitro* Assessment of NaCl to *R. sativus* (L.)

#### Germination Efficiency and Biological Attributes

*R. sativus* L. (radish) seeds were soaked for 24 h in double-distilled (dd) deionized water. Under aseptic circumstances, sodium hypochlorite (NaOCl, 1%) was used to sterilize the surface for 2 min, followed by three washing cycles with sterile distilled water. Soft agar (0.7%) plates amended with varying levels (0, 2, 5, 7, 10, 12, and 15%) of NaCl were prepared. The seeds were planted on plates of soft agar and kept at room temperature (28 ± 2°C) for 3–4 days. After 4 days, percent germination and root and shoot lengths of the plantlets were recorded.

#### Percent Survival, Tolerance Index, Cellular Permeability, and Cytotoxicity Assessment

The survival percentage of plantlets grown on soft agar plates treated with varying NaCl concentrations (0, 2, 5, 7, 10, 12, and 15%) was determined. The tolerance index (TI) was calculated by the formula used by Iqbal and Rahmati (1992).


$$\begin{array}{l} Tolerance\;indices\;\left(TI\right)\\ =\frac{Root\;length\;\left(RL\right)\;of\;NaCl\;treatment}{Root\;length\;\left(RL\right)\;of\;control\;treatment}\;\times \;100 \end{array}$$

Assessment of NaCl-induced membrane damage and cytotoxicity in root tissues of *R. sativus* (L.) was examined using confocal laser scanning microscopy (CLSM). Roots grown on NaCl-treated soft agar plates were carefully detached, cleaned, washed with phosphate buffer saline (PBS), and tagged with a fluorescently labeled dye, propidium iodide (PI: 25 μM), and observed for visually impaired dead cells (as observed with increasing red color) (Shahid and Khan, 2018; Shahid et al., 2021c). The loss of cell membrane in root tissues of salt-stressed radish seedlings was utilized as a toxicity signal to distinguish between metabolically active and inactive cells. For this, a well-adapted Evans blue staining procedure was followed (Baker and Mock, 1994).

### Isolation, Morphological and Biochemical Characterization of Recovered PGPR

For bacterial isolation, soil samples were taken from the rhizosphere soils of potato cultivated in agricultural fields with saline soils. The soil sample (refer to Supplementary Table 1 for physicochemical properties) was sieved (2-mm pore size), air dried in the shade to eliminate the excess moisture, and then utilized to isolate bacteria. The soil samples were diluted in series (serial dilution of 10^−1^ to 10^−7^), and 100 μL was spread plated over a nutrient-agar (NA) medium and incubated for 2 days at 28 ± 2°C. After incubation, bacterial colonies were purified by several times, streaking on the same medium. These isolates were subsequently examined by Gram-staining for their morphological characteristics and assessed for different biochemical tests (Holt et al., 1994).

### Selection of NaCl-Tolerant PGPR Strain and Molecular Identification of Strain KR-17

Furthermore, the NaCl tolerance ability of chosen PGPR strains was evaluated. For the assessment, all recovered isolates were grown in a nutrient broth (NB) medium added with various amounts (0–20%) of sodium chloride (NaCl) and incubated at 28 ± 2°C in a shaking incubator (at 150 rpm) for 2–3 days. Metabolically, active cells were screened using a viable count method after incubation, and the strain that showed the highest level of salt tolerance was referred to as halotolerant (salt-tolerating) PGPR strains. In addition, 16S rRNA sequencing was used to identify the isolate to the species level: *K. radicincitans* (see supplementary methods for details).

### Bioassays for Plant Growth-Regulating Substances (PGRS) Under NaCl Stress

#### Indole-3-Acetic Acid, Siderophore, and ACC Deaminase Enzyme

The modified method of Bric et al. (1991) was used to determine the synthesis of IAA by culturing the cells of *K. radicincitans* KR-17 strain in Luria Bertani (LB) broth amended with a fixed quantity of tryptophan (100 μL) and treated with varying levels of NaCl (see Supplementary Method Section S4.2a). The siderophore-producing ability of strain KR-17 was assessed by growing the bacterial cells in a salt-treated universal chrome azurol S (CAS) medium (Schwyn and Neilands, 1987; Alexander and Zuberer, 1991) (see Supplementary Method Section S4.2.b). To assess the ACC deaminase activity of KR-17 strain, the cells were grown in a liquid medium added to different salt concentrations, and the amounts of produced α-ketobutyrate were measured (Honma and Shimomura, 1978) (see Supplementary Method Section S4.2c).

#### P-Solubilization and Production of HCN and Ammonia

To estimate the P-solubilization activity, bacterial strain KR-17 was grown in Pikovskaya's (PKV) broth added with 0, 2, 5, 10, 15% NaCl, and P- solubilization activity was quantitatively evaluated. For estimation, 5 ml of culture filtrate was collected and tested for P-solubilization effectiveness, following the soluble P chlorostannous-reduced molybdophosphoric acid method Jackson, 1973. Production of the bacterial cyanogenic compounds, i.e., HCN (Bakker and Schippers, 1987) and ammonia Dye, 1962, was tested by growing the bacterial strain in an NaCl-treated/untreated HCN-induction medium and peptone water, respectively.

### Determination of Biofilm Development and Associated Traits in KR-17 Under NaCl Stress

Biofilm formation ability of bacterial strain KR-17 in absence/presence of different NaCl concentrations was assessed by growing the cells in 96 well plates and treating with 1% crystal violet (CV), following the standard procedure of O'Toole (2011) (Syed et al., 2021) (see Supplementary Method Section S2.5). Furthermore, swimming and swarming motilities of strainKR-17 were assayed (Adler, 1966). For this, spot inoculation of freshly produced cells was done on 0–15% NaCl-supplemented 0.3% and 0.5% (w/v) in nutrient agar (NA) plates and incubated at 28 ± 2°C for 2 days. After this, bacterial motilities were measured as their swarm diameter and represented in millimeters (mm). Extracellular polymeric substances (EPS) produced by KR-17 strain in the presence of salt were estimated quantitatively (Mody et al., 1989). The alginate produced by strain KR-17 was quantified. For the assay, the cells were grown in a liquid medium added with different NaCl concentrations (Wozniak et al., 2003) (see Supplementary Method Section S2.5.1). Furthermore, the cell surface hydrophobicity (CSH) of the KR-17 strain was quantified by cultivating bacterial cells with/without different NaCl concentrations using the microbial adhesion to the hydrocarbons (MATH) method (Rosenberg et al., 1983).

### Crop-Based Experiments

#### Plant Culture, NaCl Treatment, and Inoculation of Halotolerant Strain KR-11

The description for planting of *R. sativus* (L.), seed bacterization, and treatment plans for pot-house experiments has been provided in electronic supporting information (see Supplementary Method Section S2.6.1) (Khan et al., 2020).

#### Assessment of Germination Efficiency, Plant Length, Biomass and Photosynthetic Pigments

At 8 days after sowing (DAS), germination efficiency of salt-treated and bio-inoculated *R. sativus* (L.) seeds was recorded. At the harvest, plants cultivated in soils inoculated with NaCl-tolerant PGPR strain *K. radicincitans* KR-17-added plants cultivated at different salt concentrations in soils were removed, and germination efficiency and biological attributes (such as root and shoot length and weight, and dry biomass) were recorded. Accumulation of photosynthetic pigments (chlorophylls and carotenoids) in the NaCl-treated/untreated and bacterized plants was measured using universal methods of Arnon (1949) and Kirk and Allen (1965).

#### Estimation of Protein and Carbohydrate Contents in Root Tissues of *R. sativus* (L.)

The protein (AOAC, 1990) and carbohydrate contents (Prud'homme et al., 1992) accumulated in root tissues of NaCl-treated and PGPR inoculated *R. sativus* plants were estimated.

#### Carotenoids and Flavonoids Estimation in *R. sativus* (L.) Root Tissues

The carotenoid contents accumulated in roots tissues of NaCl-treated and PGPR-inoculated *R. sativus* (L.) were extracted (Rodriguez et al., 1976). For this, 5-g freshly detached root samples were homogenized in 80% acetone. The acetone layer was separated, and 20 ml of the petroleum ether was added. At the separation stage, H_2_O was mixed, whereas, at the end of separation, the petroleum ether-carotenoid phase is made a volume of up to 50 ml. This petroleum-ether extract was spectrophotometrically (at 450 λ) determined. Carotenoid content was estimated and expressed as β-carotene (g ml^−1^) and calculated as:


$$\begin{array}{l} \beta -carotene=A\times df\times V/E1\%1cm\times w \end{array}$$

where A, df, E1%, w, and V represent absorbance, dilution factor, absorbance co-efficient (2,592 for petroleum ether), 1 cm, weight of sample (g) and volume (ml), respectively.

The total flavonoid content in roots of *R. sativus* (L.) was estimated by adopting the method of Sultana et al. (2009). For estimation, 10-g freshly detached roots were homogenized in methanol with the help of an electric blender. To the filtrate, 1 ml was taken and 4 ml of water was added to this. At the start, 0.3 ml of 5% NaNO_2_ solution was mixed with them. After 5 min of incubation, 10% AlCl_3_ (w/w) was mixed, and, after 6 min, 2 ml of 1-M NaOH was added to the solution. The absorbance was read at 430 nm.

#### Estimation of Root Ascorbic Acid and Lysine Content

Ascorbic acid (AsA) contents in freshly removed root tissues of KR-17-inoculated and NaCl-treated *R. sativus* (L.) plants were estimated (Mukherjee and Choudhuri, 1983) (see Supplementary Method Section S2.6.5). To determine the lysine content in inoculated and salt-treated *R. sativus* (L.) roots, we applied the previously described method of Galicia et al. (2009).

#### Determination of Mineral Composition in Root Tissues of *R. sativus* (L.)

The mineral content accumulated in salinity-stressed and bio-inoculated root tissues of radish was determined. For estimation, 0.1 g of root samples was oven-dried following acid digestion. Mineral contents like sodium (Na), calcium (Ca), potassium (K), magnesium (Mg), iron (Fe), zinc (Zn), copper (Cu), phosphorous (P), and nitrogen (N), etc., were determined using spectrophotometer and atomic absorption a spectrophotometer (AAS) (see Supplementary Method Section S2.6.6).

#### Estimation of Total Lipids, Phenolic Compound, and Alkaloid in *R. sativus* (L.)

Total lipid contents accumulated in salt-treated and bio-inoculated *R. sativus* (L.) leaves were measured quantitatively using chloroform and methanol in the ratio of 2:1 (Bligh and Dyer, 1959). Furthermore, accumulation of total phenolic content in NaCl-treated and PGPR-inoculated radish foliage was analyzed using the method of Jindal and Singh (1975). The spectrophotometric analysis was applied to quantify the total alkaloid content in NaCl-supplemented and salt-alleviating PGPR-inoculated radish foliage.

#### Assessment of Membrane Injury and Oxidative Stress Under Saline Stress

##### Assessment of Membrane Damage and Relative Leaf Water Content

Freshly detached NaCl-untreated/treated and PGPR-inoculated leaves of *R. sativus* (L.) were used to determine the membrane damage and relative leaf water content (RLWC). The leaf electrolyte leakage (EL) was used to evaluate membrane injury/damage. For this, 1 g of foliage was placed in a vial containing 10-ml sterilized H_2_O and incubated for 1 day at 25°C, following which electrical conductivity (EC) of solution (L1) was determined. After that, the samples were placed in a boiling water bath (120°C) for 30 min, and EC (L2) was determined. Electrolyte leakage (percentage) was estimated (Lutts, 1996):


$$\begin{array}{l} Electrolyte\;leakage\;\left(\%\right)=L1/L2\times 100 \end{array}$$

In order to determine relative leaf water content (RLWC), the method of Barrs and Weatherley (1962) was applied. For this, leaf samples were cut in pieces, weighed and maintained for 3 h in DDW to get turgid weight. The samples were then oven-dried (at 80°C) for 24 h until constant weight. The RLWC was calculated as:


$$\begin{array}{l} RLWC\left(\%\right)=\frac{FW-DW}{TW-DW}\;\times \;100 \end{array}$$

Here, FW = fresh weight, DW = dry weight, TW = turgid weight

##### Estimation of Free Proline in *R. sativus* (L.)

The free proline content accumulated in roots and foliage of *R*. *sativus* (L.) plants cultivated with and without the amendment of NaCl and inoculated with halotolerant KR-17 strain was assayed as demonstrated earlier (Bates et al., 1973) (See Supplementary Method Section S2.6.8.2).

##### MDA Content (Lipid Peroxidation) Estimation

Lipid peroxidation (MDA content) in roots and foliage of *R*. *sativus* (L.) cultivated with/without the amendment of NaCl and inoculated with halotolerant KR-17 strain was calculated as malondialdehyde (MDA). Absorbance of abduct (MDA-TBA2) formation after reaction of thiobarbituric acid (TBA) and MDA was spectrophotometrically measured (Heath and Packer, 1968). In order to assess the MDA content, 500 mg of fresh roots and foliage was homogenized with 10.-ml tri-chloroacetic acid (TCA; 5% w/v) on an ice bath, followed by centrifugation (12,000 × *g*) for 20 min (at 4°C temperature). In clean acid-washed glass tubes, equal quantities of the resultant supernatant and thiobarbituric acid (TBA;0.67 percent w/v) were combined, and this mixture was heated (at 100°C) for a period of half an hour. To stop the reaction, it was placed in an ice bath. After centrifugation (10,000 × *g*) at 4°C for 10 min, optical density (OD) of supernatant was recorded at three wavelengths (λ) of 450, 532, and 600. The MDA levels were calculated using equation and molar extinction coefficient of 155 mM^−1^ cm^−1^.


$$\begin{array}{l} MDA\;level\left(\mu mol/L\right)=6.45\times \left(A\lambda 532-A\lambda 600\right)-0.56\times A\lambda 450\\ \;where\;A=absorbance \end{array}$$

#### Extraction and Determination of Antioxidant Enzymes

The antioxidative defense enzymes like ascorbate peroxidase (APX), catalase (CAT), superoxide dismutase (SOD), and glutathione reductase (GR) of salt-treated and KR-17 strain inoculated in radish foliage were determined (See Supplementary Method Section S2.6.8).

#### Determination of *in vitro* Biofilm Formation

The ability of strain KR-17 to produce biofilms in the presence of NaCl was tested in polystyrene wells of a 96-well micro titer plate and on a glass surface (Ahmed et al., 2021). In a nutshell, a young culture (1 × 10^6^ CFU ml^−1^) of KR-17 strain was inoculated in NB broth added with glucose (5%) and NaCl. Following growth, next stages were the same as those outlined by Ahmed et al. (2021). Then, microscopic examination was done.

#### Characterization for *in vivo* Biofilm Formation and Further Colonization

The 45-day-grown NaCl-untreated/treated *Raphanus sativus* (L.) roots were surface sterilized and treated with 24-h old culture (1 × 10^7^ CFU ml^−1^) of *K. radicincitans* KR-17. Surface sterilization efficiency of roots was checked by last wash plating and tissue imprinting. The roots (both untreated and treated with varying levels of salts) were dipped in the bacterial inoculum for a period of 12 h and allowed for biofilm formation. After that, the roots were washed with phosphate-buffered saline (PBS) thoroughly and cleaned with double-distilled water. The root samples were then processed for scanning electron microscopic (SEM) observation for checking the observation of biofilm formation over plant roots and further colonization.

#### Statistical Analysis

Three replications (each treatment) of the trials were conducted in complete randomized block design (RBD). Sigma Plot 12.0 and Minitab 17.0 software was used to do statistical analysis of experimental data. Tests included two-way ANOVA, followed by *post hoc* least significant difference (LSD). Student's *T*-test was used to compare the data at *p* ≤ 0.05, *p* ≤ 0.005, and *p* ≤ 0.001 levels. The statistical software Sigma 13.0 was used to prepare the graphs.

## Results and Discussion

### Effect of NaCl on *Raphanus sativus* (L.) Seedling: *In vitro* Studies

#### Seed Germination, Vigor Index, and Plant Length of *R. sativus* (L.)

**Figure 1:**
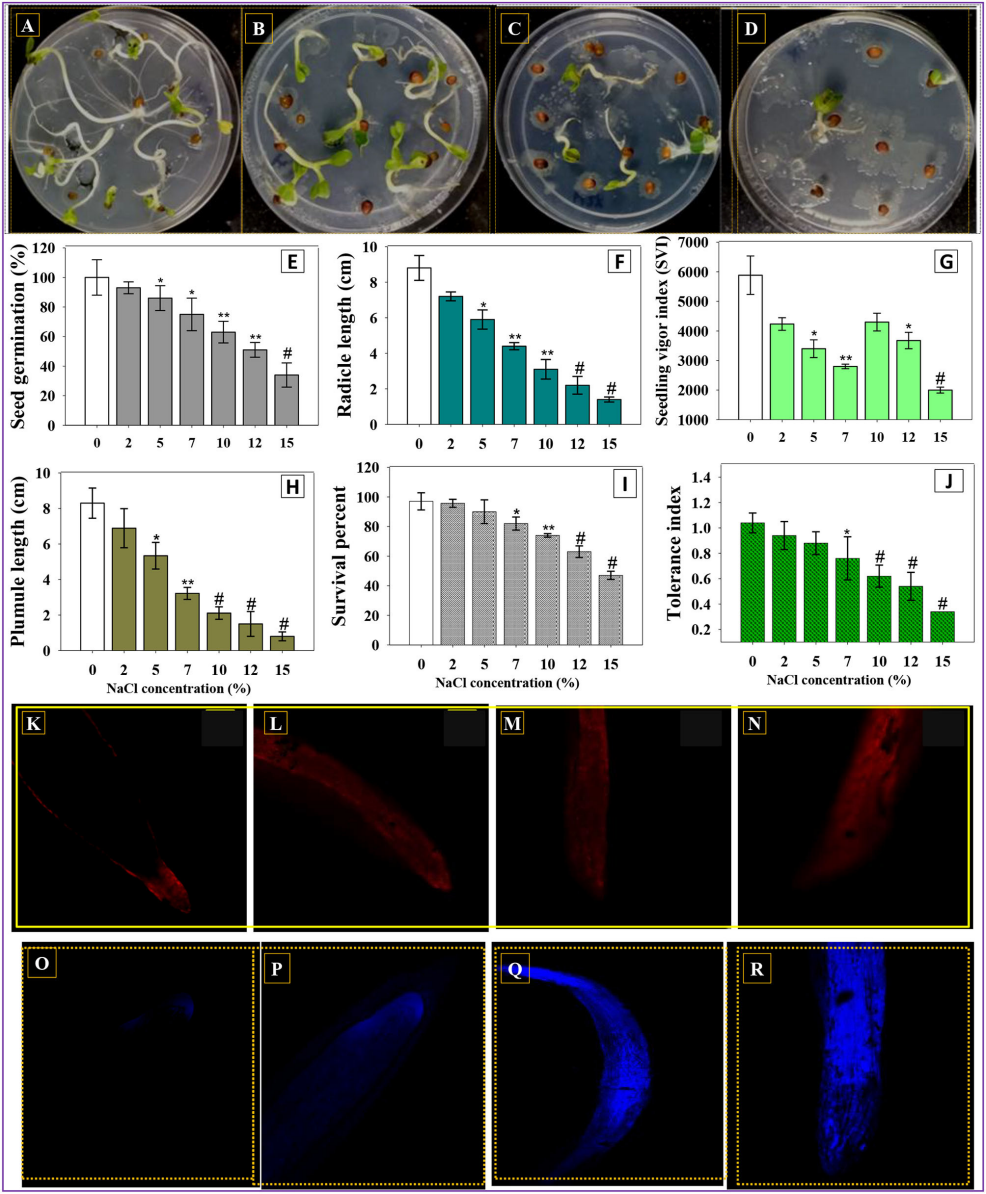
*R. sativus* seeds germinated on soft agar plates treated with 0% **(A)**, 5% **(B)**, 10% **(C)**, and 15% NaCl **(D)**. Effect of different rates of NaCl on germination efficiency **(E)**, radical length **(F)**, vigor index **(G)**, plumule length **(H)** survival **(I)**, and tolerance index **(J)** of *R. sativus* seeds grown on soft agar plates *in vitro*. **(K–R)** represent the CLSM images of NaCl-induced permeability and cellular death in root tissues of radish treated with 7, 10, 12, and 15% NaCl, respectively. In this figure, bar diagrams represent the mean values of three replicates (*n* = 3). Corresponding error bars represent standard deviation (S.D) of three replicates (SD, *n* = 3). The asterisks *, **, and # denote statistical significance at *p* < 0.05, *p* < 0.005 and *p* < 0.001, respectively, computed by Student's *T*-test.

In order to assess the salinity-induced negative impact on crop plants, seeds of *R. sativus* (L.) (radish) were sown on plates of soft agar, added with different levels (0, 2, 5, 7, 10, 12, and 15%) of NaCl under *in vitro* condition. The obtained result demonstrated that a higher level of NaCl (15%) had a maximum detrimental effect on characteristic features of germinated radish seedlings (Figures 1A–D). For instances, at 15% NaCl, germination efficiency (*p* < 0.001) (Figure 1E), seedling vigor index; SVI (*p* < 0.001) (Figure 1F), radical length (RL) (*p* < 0.001) (Figure 1G), and plumule length (PL) (*p* < 0.001) (Figure 1H) of radish seedlings were drastically reduced by 66, 65, 84, and 90%, respectively, over control treatment (without any salt). In the life cycle of plants to be cultivated, germination is a complex biological process that requires several variables to work together for a seedling to develop. Germination vigor is determined by the ability/capacity of plant embryo to resume the metabolic process in a coordinated and sequential way after being implanted within the seed. The reduced germination and vigor indices observed in this study represented that the NaCl-induced osmotic barrier affects the water uptake, which prevents the seed water uptake by generating an external osmotic potential, and, thus, reduction in a germination attribute occurs. Under *in vitro* circumstances, various concentrations of NaCl detrimentally affected the germination efficiency, vigor indices, and biological features of water spinach (*Ipomoea aquatica*) (Ibrahim et al., 2019).

#### Plant Survival, Tolerance Index, Root Membrane Permeability, and Cell Death

The survival percent of *R. sativus* (L.) seedlings grown on soft agar plates was varied. The higher the level of NaCl, the greater negative effect on seedling survival percentage is. For instance, 15% NaCl concentration maximally decreased (51%) the seedling survival over control (Figure 1I). Likewise, tolerance indices (TI) in *R. sativus* (L.) were significantly reduced as the salt levels were increased, confirming a negative relationship between NaCl and TI. The *R. sativus* (L.) tolerance index was measured up to 1.04, 0.94, 0.88, 0.76, 0.62, 0.54, and 0.34 at 0, 2, 5, 7, 10, 12, and 15% NaCl levels, respectively (Figure 1J). These findings revealed that the lower level of salts had the highest TI, whereas greater NaCl concentrations had the lowest tolerance indices. Similarly, the tolerance of varying levels of NaCl in *S. lycopersicum* L. (tomato) germplasm at seedling stages has been reported (Rehman et al., 2019).

Additionally, the root membrane integrity of salt-treated *R. sativus* was examined using confocal laser (CLSM) microscopy to better analyze the detrimental impact of NaCl on root membrane. Propidium iodide (PI), a DNA-binding dye, was, therefore, utilized to differentiate between viable/active cells and cells with damaged/disrupted root membrane, following exposure to varying levels of NaCl. Surprisingly, the roots treated with different concentrations of NaCl showed a concentration-dependent increase in dead/injured cells (Figures 1K–N). Damage/injuries to the integrity of cell membranes may be associated with morphological alterations in cells (Ziegler and Groscurth, 2004). With higher NaCl concentrations and longer treatment times, the harmful impact became more pronounced. Nucleotides may be created by DNA degradation and reallocated for shoot and new root forms when salt stress is transitory or modifiable at the seedling stage. Following salt treatment, cell death was detected in this investigation. The growth of plant roots could be slowed as a result of cell death. A high NaCl concentration can significantly limit the plant seedling growth, almost totally stopping it. Similarly, NaCl-induced cytotoxicity was observed by staining the NaCl-untreated/treated root samples with Evan's blue dye (acidic and non-permitting exclusion). Here, Evan's blue-stained NaCl-treated roots showed a concentration-dependent increase in the uptake of the dye, confirming the cellular death in root tissues of plants (Figures 1O–R). This blue dye can penetrate thru the ruptured/destabilized cell membrane.

### Isolation of PGPR, Salt Tolerance, and Molecular Identification

In current agricultural techniques, salinity is a serious issue. It has a significant negative impact on the growth, biological characteristics, and yield attributes of various important edible crops. To address these issues, we attempted to identify a salt-tolerating rhizosphere PGPR strain that might be used as a microbial inoculant to boost the growth and yield of crops raised in salty environments. During the investigation and isolation of microbial strains for NaCl stress relief, a total of 15 PGPR isolates were recovered from the vegetable rhizosphere cultivated in a saline environment and tested for their morphological and biochemical characteristics. All of the strains were detected under a light microscope as red/pink-colored short rods (Gm –ve), with a varied response to different biochemical assays. In this study, strain KR-17 survived an exceptionally high level of salt concentration (18% NaCl; 3-M concentration) (Supplementary Table 2). Strain KR-17 was chosen for agricultural trials because of its high salt tolerance profile. This strain exhibited a varied level of biochemical reactions (Supplementary Table 3). The KR-17 strain was part of the genus *Kosakonia* on the basis of morphological, biochemical (citrate utilization, indole production, NO_3_- reduction, oxidase, catalase, starch, gelatin, dextrose, and glucose, etc.), and cultural (an irregular margin; a mucoid colony; optimum temperature, 30°C; pH 7.0; no any pigmentation) features. In addition, this strain was identified molecularly by 16S rRNA gene sequencing for species-level identification. The 16S rRNA nucleotide sequences (1,085 bp) of strain KR-17 were submitted to GenBank (Accession No. OM348535). The BLASTn tool was used to do a similarity search, which revealed that strain KR-17 was closely related to *Kosakonia radicincitans* due to its highest relatedness. Then, MEGA 7.0 software was used to create a phylogenetic tree (Supplementary Figure 1) based on the 16S rRNA partial gene sequences retrieved from the NCBI portal. Likewise, numerous workers recovered halotolerant PGPR strains *viz*., *Kocuriarhizophila* (Li et al., 2020); *Alcaligenes faecalis* (Babar et al., 2021); *Kosakonia sacchari* (Shahid et al., 2021a); *Bacillus amyloliquefaciens* and *B. pumilus* (Sharma et al., 2021); *Achromobacter denitrificans* and *Ochrobactrum intermedium* (Sultana et al., 2020) identified them based on 16S rRNA sequencing.

### Essential PGP Substances of *K. radicincitans* Under NaCl Stress

#### Indole-3-Acetic Acid and 1-Amino Cyclopropane 1-Carboxylate Deaminase

**Figure 2:**
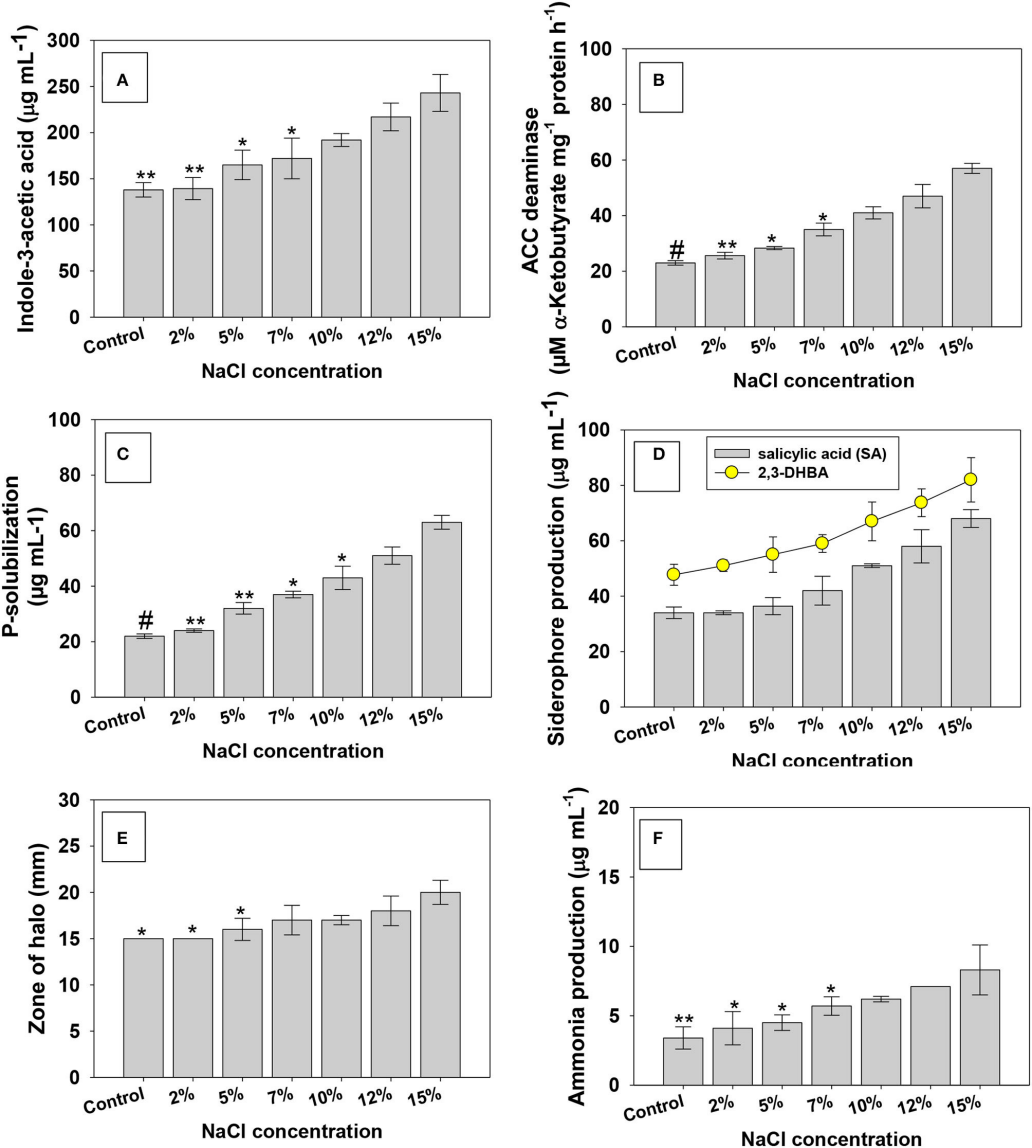
Influence of different levels of the NaCl (0, 2, 5, 7, 10, 12, and 15%) growth-regulating substance secreted/released by PGPR strain under *in vitro* condition; indole-3-acetic acid **(A)** ACC deaminase **(B)**, P-solubilization **(C)**, siderophore production **(D,E)**, and ammonia production **(F)**. In this figure, bar and line diagrams represent the mean values of three replicates (*n* = 3). Corresponding error bars represent standard deviation (S.D) of three replicates (SD, *n* = 3). The asterisks *, **, and # denote statistical significance at *p* < 0.05, *p* < 0.005, and *p* < 0.001, respectively, computed by Student's *T*-test.

Here, salt-tolerating PGPR strain KR-17 when grown in the absence (Supplementary Table 4)/presence of salt-stressed environment, production of bioactive molecules (growth-controlling chemicals) was uneven. With increasing NaCl concentrations, growth-regulating activities of bacterial strain were increased. Strain KR-17 produced 138 ± 7.8 μg IAA ml^−1^ at 0% NaCl, which yet increased with graded NaCl concentrations. For instance, a maximum of 243 ± 20 μg IAA ml^−1^ (43% increase over control) was recorded at a higher (15% NaCl) concentration (Figure 2A). The increased quantum of IAA in the presence of increased salinity levels is related to the salt tolerance capacity of the bacterial strain. It has been reported that 80% of rhizobacterial populations release indole-3-acetic acid (IAA), a physiologically active auxin (Park et al., 2021). Even under harsher settings, IAA produced by soil microorganisms regulates cell development and proliferation, root morphogenesis, apical dominance, phototropism, and other physiological activities. In addition, IAA loosens the cell walls of roots, resulting in a reduction in root exudates, which stimulates the development of PGPR by supplying extra nutrients (Glick, 2012). Several IAA-producing halotolerant PGPR strains residing inside the salinity-stressed environments have been shown to improve the growth of leafy vegetables under salty conditions. In a study, Ahmad et al. (2013) reported that indole-3-acetic acid produced by halotolerant *Rhizobium* and *Pseudomonas* strains enhanced the overall performance of plants under salt stress. Similarly, a considerable increase in IAA production with increasing NaCl is reported (Hidri et al., 2019). Even at greater levels of NaCl, halotolerant KR-17 strain secreted IAA, which is an unusual and encouraging characteristic of halotolerant microbes, because such halotolerant PGPR strains are more likely to endure synthesizing indole-3-acetic acid and allowing plants to access this important growth-promoting phytohormone even in the salty environment (Mehmood et al., 2018).

The 1-amino cyclopropane 1-carboxylate (ACC) deaminase produced by a variety of plant-beneficial soil microorganisms is another exceptional biological characteristic that may significantly reduce the ethylene levels in plants and, hence, speed up the functioning of growing plants under harsher environments (Gao et al., 2020; Jha et al., 2021). Here, even when grown in media supplemented with different levels (0, 2, 5, 7, 10, 12, and 15%) of NaCl, strain KR-17 showed a favorable response to ACC deaminase. The amount of α-ketobutyrate produced by *K. radicincitans* has steadily increased with increasing levels of salt. For example, at 15% NaCl, strain KR-17 produced 57 ± 1.8 μM α-ketobutyrate mg^−1^ protein hour^−1^ (>59% over untreated control) (Figure 2B). Several salt-tolerant bacterial strains utilizing ACC as their only source of nitrogen (N) have been identified. Plants often produce ethylene as a stress response, and it is closely linked to different stress conditions, such as drought, salt, metal toxicity, and nutrient shortage (Riyazuddin et al., 2020). As a result, ACCD produced by PGPR shields the plants from the damaging effects of ethylene when they are exposed to abiotic stresses (Sapre et al., 2019). Several researchers have found that halotolerant PGPR strains exhibited the production of ACC deaminase when exposed to a harsher saline environment (Wu et al., 2012; Sarkar et al., 2018; Ji et al., 2020; Han et al., 2021). Like our study, salinity-alleviating *Bacillus* sp. has been reported to alleviate the salt stress and promoted the growth of *Zea mays* L. (maize) by expressing ACC deaminase exogenously (Misra and Chauhan, 2020). A large number of ACC deaminase positive PGPR have also been shown to aid the development of vegetables in derelict/stressed soils, in addition to their involvement in crop improvement in traditional soil.

#### P-Solubilizing Activity and Siderophore Production

Under increasing NaCl concentration, a similar pattern of increase as with other measured PGP substances in P-solubilization was observed (Figure 2C). The second most important plant nutrient after water is phosphorous, P (a master key element), and it is involved in virtually all metabolic processes of plants, including genetic transmission, chlorophyll production, respiration, signaling molecule transduction, and energy transfer (Billah et al., 2019). Plant development is greatly hampered by a shortage of phosphorous. Plants have access to <5% of total soil P (Dobbelaere et al., 2003). As a result, phosphate fertilizers are administered from outside sources to avoid P deficiency and allow plants to develop properly. In this regard, P-solubilizing microorganisms (PSM) of various genera have provided some alternatives to costly synthetic P fertilizers (Alori et al., 2017). Several halotolerant PGPR, including *Kocuriarhizophila* (Li et al., 2020), *Bacillusmegatrium* (Akcay and Kaya, 2019), *Enterobacter asburiae* (Mahdi et al., 2020), *Acinetobacter, Pseudomonas* (Jiang et al., 2020), etc., has been reported to exhibit the P-solubilizing activity at greater NaCl concentrations. These halotolerant PSBs are reported to increase salt-tolerance levels in various vegetable crops. Microbes use a variety of ways to provide P to plants, (i) phosphate solubilization: organic acids, OH– ions, CO_2_, and protons are produced and released, respectively (ii) biochemical phosphate mineralization: catalyzed by the release of extracellular enzymes.

In Fe-deficient situations, siderophore, aniron (Fe)-chelating complex, with a low molecular weight produced by soil microbial diversity, transports Fe to plants (Garg et al., 2021). Insoluble iron is divided into two types; (i) hydroxide and (ii) oxyhydroxide that become unavailable to rhizobacteria (Bonneville et al., 2004). As a result, the release of siderophore in the presence of Fe deficiency might be beneficial since a PGPR strain capable of producing siderophore might be utilized in the biological management of plant diseases. Like other growth-regulating traits, increasing NaCl concentration caused a pronounced increase in bacterial production of siderophore. For example, under controlled conditions, strain KR-17 produced 34 μg ml^−1^ of salicylic acid (SA), which, however, increased by 50% at 15% NaCl concentration (Figure 2D). A trend similar was recorded for halo formation (a siderophore zone) under NaCl stress (Figure 2E). Under iron-limiting circumstances, a siderophore largely aids the producing organism in iron acquisition. The Fusarium-wilt disease in pepper is caused by *F*. *oxysporum Schl*. *f*. sp. *capsici* is controlled by siderophore generated by *B. subtilis* CAS15 strain (Yu et al., 2011). Similarly, *B. amyloliquefaciens* produced a considerable amount of siderophore, which helps to prevent the tomato crop from bacterial wilt disease (Singh et al., 2015). Similar to our work, enhanced synthesis of siderophore was also observed at increased NaCl levels (Panwar et al., 2016).

#### HCN and NH_3_ Production

The concentration of NaCl had no effect on production of ammonia (Figure 2F) and cyanogenic (HCN) compound (Supplementary Table 5) produced by KR-17 strain. Another metabolite generated by a high bacterial population is ammonia, which is created by the breakdown of amino acid and ammonification of nitrite, hydrolytic-mediated urea, and decarboxylation of amino acid. Furthermore, many PGPR strains include ammonia transporters within their cells that are believed to participate in NH4^+^ absorption due to NH_3_ diffusion across the bacterial cell membrane (Patriarca et al., 2002). Like this finding, a number of HCN and ammonia-producing halotolerant PGP bacterial strains are reported to improve the growth attributes in several edible crops by increasing their tolerance levels to salt stress (Goswami et al., 2014; Kerbab et al., 2021; Shahid et al., 2021a).

### Development of Biofilm and Associated Traits (EPS Production, Swimming and Swarming Motility) Under Salinity Stress

Impact of different levels of NaCl on biofilm formation, cell adhesion ability to hydrocarbons, and motility of *K*. *radicincitans* KR-17 was evaluated *in vitro*. Here, formation of bacterial biofilm was increased with increasing NaCl. For example, a 61% increase in biofilm development was noticed at a higher level of salt over control (Figure 2A). Similarly, effectiveness of biofilm-forming and EPS-producing abilities of PGPR strains under saline stress has been reported. Understanding the biological implications of exopolysaccharides (EPS) synthesized by a variety of soil bacterial populations, the impact of NaCl on EPS produced by halotolerant KR-17 strain was evaluated. In the absence of salt, KR-17 strain produced a considerable amount (167 μg ml^−1^) of EPS. Interestingly, quantum of EPS was increased as the level of NaCl was increased. For instance, at 15% NaCl, the quantum of produced EPS was 60% higher as compared to control (Supplementary Figure 2B). EPS are water-in-polymer-matrix-hydrated molecules that provide immediate protection against desiccation in developing seeds. It has been reported that bacterial production of EPS and alginate is likely to improve the survival strategy and enhance the rate of production of active metabolites under harsher conditions, such as drought, salinity, and others (Egamberdieva et al., 2019). Likewise, Upadhyay et al. (2011) found that four salt-resistant PGPR strains secreted EPS continuously in a growth medium supplemented with increasing NaCl concentrations. Furthermore, inoculating *Vicia faba* L. (faba bean) plants with EPS-synthesizing and biofilm-forming halotolerant PGPR *Pseudomonas anguilliseptica* SAW-24 strains increased the growth features of crops against increasing salt concentrations (Alaa, 2018). Similarly, swimming and swarming motilities of KR-17 strain showed a significant increase with rising NaCl concentrations. In the absence of salt (at 0% NaCl), the swarming and swimming motility of KR-17 strain was recorded as 31 mm and 21 mm, which, however, maximally increased by 28 and 43%, respectively, at higher concentration (15% of NaCl) (Supplementary Figures 2C,D).

#### Alginate Production and Cell Surface Hydrophobicity

The production of alginate by strain KR-17 was diminished with increasing NaCl concentration. The minimum production (74 μg ml^−1^) of alginate was recorded at 15% salt concentration (Supplementary Figure 2E). Adhesion of bacterial cells to hydrocarbons or ability of cell surface hydrophobicity (CSH) of *K*. *radicincitans* KR-17 was also reduced with an increasing level of NaCl stress. For example, 15% NaCl concentration, CSH of strain KR-17 was maximally reduced by 58% over control (Supplementary Figure 2F). Cell surface hydrophobicity in bacterial species is associated with bacterial cell aggregation and adhesion, as well as biofilm formation. As a result, excessive levels of salinity stress on bacterial cells may impede the bacterial species colonization behavior. The continued formation of biofilm and production of associated traits even at higher concentrations of NaCl is a clear indication that salt-tolerating PGPR strain has the ability to withstand even under harsher salty environment. Salt tolerance is predicted to provide protection to the developing bacterial cells, as well as increase the survival and activity of halotolerant PGPR in stressful situations.

### Plant-Microbe Interaction Under Salinity Stress: Pot-House Studies

#### Bio-Inoculation of Halotolerant *K. radicincitans* KR-17 Positively Affected the Germination, Efficiency, Vigor Index, Growth, Biomass, and Chlorophyll Content of NaCl-Treated Radish

**Figure 3:**
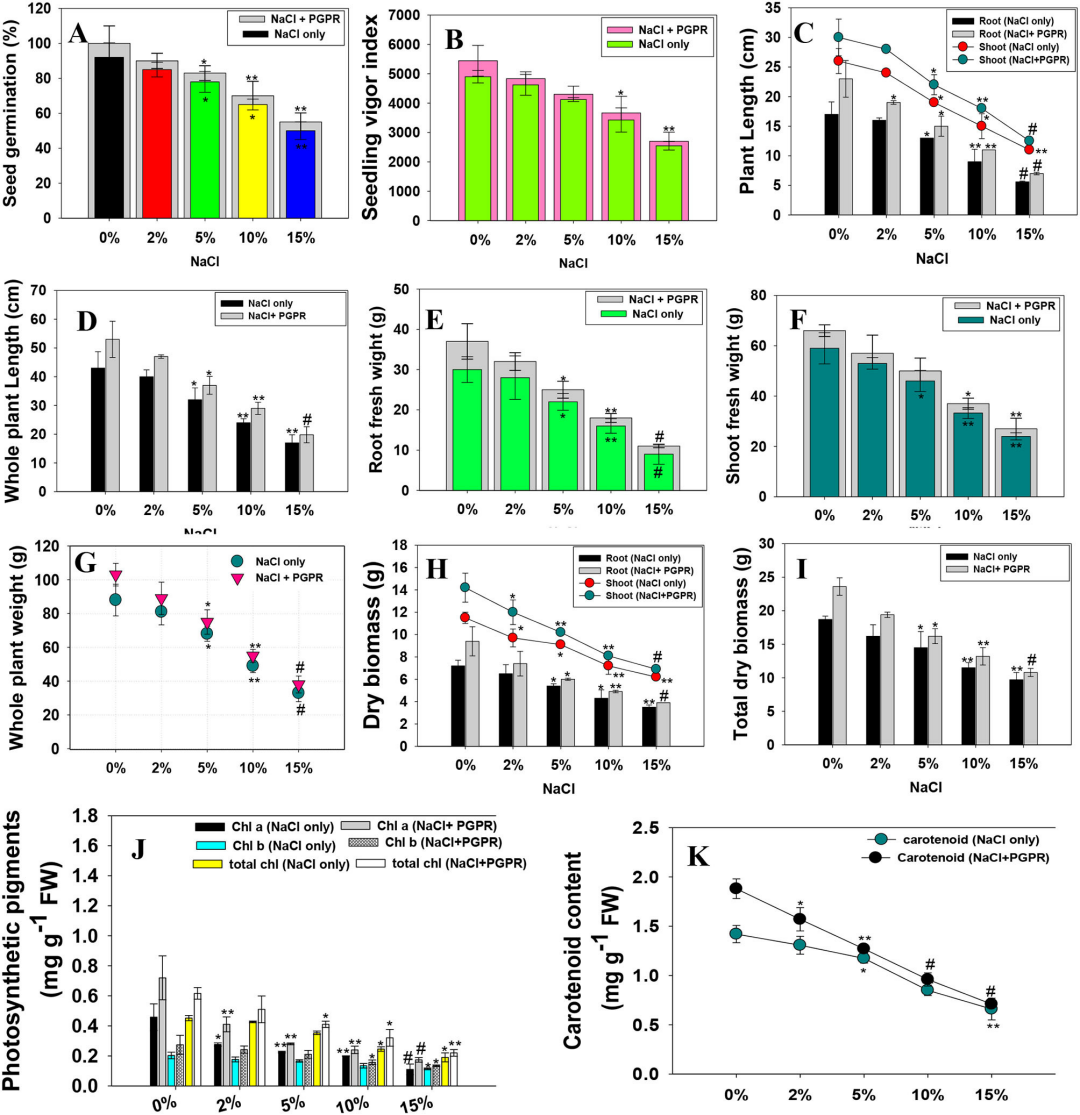
Bio-inoculation impact of halotolerant PGPR strain on germination efficiency **(A)**, Vigor index **(B)**, Biological attributes; root shoot length **(C)**, Whole plant length **(D)**, Root **(E)**, Shoot fresh weight **(F)**, Whole plant weight **(G)**, Root-shoot dry biomass **(H)**, Total dry biomass **(I)**, Photosynthetic pigments **(J)** and carotenoid content **(K)** of radish plants raised in soils treated with different levels of NaCl. In this figure, the bar, and line diagrams represent the mean values of three replicates (*n* = 3). Corresponding error bars represent the standard deviation (S.D) of three replicates (S.D, *n* = 3). The asterisks *, ** and # denote statistical significance at *p* < 0.05, *p* < 0.005 and *p* < 0.001, respectively computed by Student's *t*-test.

The ability of seeds to germinate in saline circumstances may appear to be straightforward and helpful criteria for selecting NaCl-tolerant microbial populations. Furthermore, salt stress has a greater impact on germinating seeds and seedlings than on growing plants, because germination occurs in surface soils, which collect soluble salts as a result of evaporation and a capillary rise in soil water content. High salt concentrations reduce the amount of water available to geminating seeds and seedlings, damage the structure of different enzymes and macromolecules, and halt protein metabolism, respiration, and chlorophyll formation. When planted in inoculated and untreated control soils, almost all of the seeds germinated. However, higher salt concentration (15% NaCl) maximally reduced the seed germination ability and vigor indices of *R. sativus* (L.). However, salt-alleviating PGPR strain improved the germination efficiency and vigor index when applied to NaCl-treated radish plants. For instance, strain KR-17 increased the percentage of germination and vigor index of radish plants to the greatest extent possible in the presence of 2% NaCl (Figures 3A,B). Seed priming with halotolerant PGPR had beneficial effect in the form of higher germination, as well as improved physio-biochemical characteristics (Kasim et al., 2016). This increased tolerance also enables the seeds to deal with other environmental challenges, resulting in the enhanced seedling establishment. The colonization of biofilm-forming, IAA and EPS-secreting halotolerant PGP bacterial communities around the germinating seeds and seedling roots under salt stress could be the possible reason of growth enhancement. In addition, inoculated bacteria also promoted the production of plant hormones (for example, indole-3-acetic acid), which directly stimulate the activity of enzymes (for example, amylase). This resulted in an increase in starch absorption, which encourages the early germination even in stressful situations. Improvements in auxin (IAA) synthesis by PGPR would result in a significant boost in seedling vigor. Like this study, salinity-alleviating *Pseudomonas putida* strain Rs-198 increased the germination efficiency of salt-stressed cotton seedlings (Yao et al., 2010).

Under the pot-house condition, bio-inoculated but NaCl-treated plants had varied growth conditions. The growth characteristics were generally reduced, with an increase in concentrations of NaCl, which, however, increased after soil application of strain KR-17 over un-inoculated plants. As an example, when treated with 15% NaCl concentration, roots (RL), shoots (SL), and whole plant length (WPL), roots (RB), shoots (SB) and total dry biomass (TDB) were greatly and negatively impacted. In the presence of NaCl stress, however, a steady increase in bacterized plants was observed. For instance, strain KR-17 has shown beneficial effects on the determinant of plants and enhanced the growth and dry weight even at high concentrations of NaCl (Figures 3C–I). The continued production of IAA in the presence of increasing levels of NaCl is likely to be responsible for growth promotion of plants, which benefits them in a variety of ways, including root morphogenesis. Like other stress-tolerant PGPR, salt-tolerant *K. radicincitans* strain KR-17, which was used as a strong salt reliever in the current study, generated a significant increase in the overall functioning of *R. sativus* (L.), which could be attributable to the release of bioactive molecules by beneficial soil bacteria (Shahid et al., 2021b). IAA, for instance, among these bio-stimulants directly provoke the plant root development (Figure 3J) by different physiological processes, such as cell division, elongation of cells, morphogenesis, and apical prevalence (Duca et al., 2018). As a result, enlarged roots take more water and nutrients from the soil, resulting in stronger plants. Plant growth is also influenced by other growth-regulating variables like available phosphorous, production of siderophore, ACCD, cyanogenic compounds, and ammonia. Likewise, NaCl-tolerating *B. subtilis* enhanced the growth and biomass of *Triticum aestivum* L. (wheat) crop cultivated in NaCl-stressed soils (Jabborova et al., 2020). Furthermore, NaCl-tolerant and ACCD-synthesizing *Bacillus* strains WU-13 increased the fresh weight, dry biomass, root and shoot length of *Capsicum annuum* L. (pepper) seedlings raised under saline environment (Wang et al., 2018).

In this work, formation of chlorophyll in bacteria inoculated and NaCl-stressed plants decreased with increasing NaCl concentrations. For example, higher concentration of NaCl significantly reduced the total chlorophyll and carotenoid pigments of *R. sativus* (L.). Salt stress has a negative impact on the photosynthetic system, resulting in reduced synthesis of carotenoid and chlorophyll, owing to enzyme degradation that is responsible for the development and synthesis of pigments inside the leaf tissues (Sharma et al., 2020). In contrast, at 2% NaCl, strain KR-17 increased the chl a, chl b, total chlorophyll, and carotenoid content by 34, 29, 18, and 17%, respectively, over un-inoculated but treated with a similar level of NaCl (Figure 3K). The reason behind the increase in the leaf pigments under salinity stress is halotolerant PGP bacteria might possibly promote the antioxidants and polyamines in salt-affected plants, resulting in increased photosynthetic efficiency. The root biomass and total chlorophyll content have shown a high association during the calculation of the correlation (*R*^2^ = 0.91). Also, total dry biomass and carotenoid were positively correlated (*R*^2^ = 0.89). Similar to our study, *Kocuriarhizophila* Y-1, a novel halotolerant PGPR, increased the tolerance of NaCl in *Zeamays* L. (maize) plants by adjusting the levels of phytohormone, nutrient intake, ionic homeostasis, and photosynthetic capability (Li et al., 2020). In addition, inoculation of salt-resistant *B. subtilis* improved chlorophyll production in *Bassia indica* (L.) plants cultivated in the presence of high concentration of salts (Abeer et al., 2015). Additionally, single or combined inoculation of salt-tolerating bacterial strains *Pantoea ananatis* and *Piriformospora indica* has shown to improve the chlorophyll and carotenoid molecules in rice (Gilani et al., 2018).

#### Halotolerant *K. radicincitans* KR-17 Improved the Total Root Yield, Amino Acids, and Pigment Composition of Salinity-Stressed Radish

##### Total Root Protein, Carbohydrate, Carotenoids, and Flavonoids

**Figure 4:**
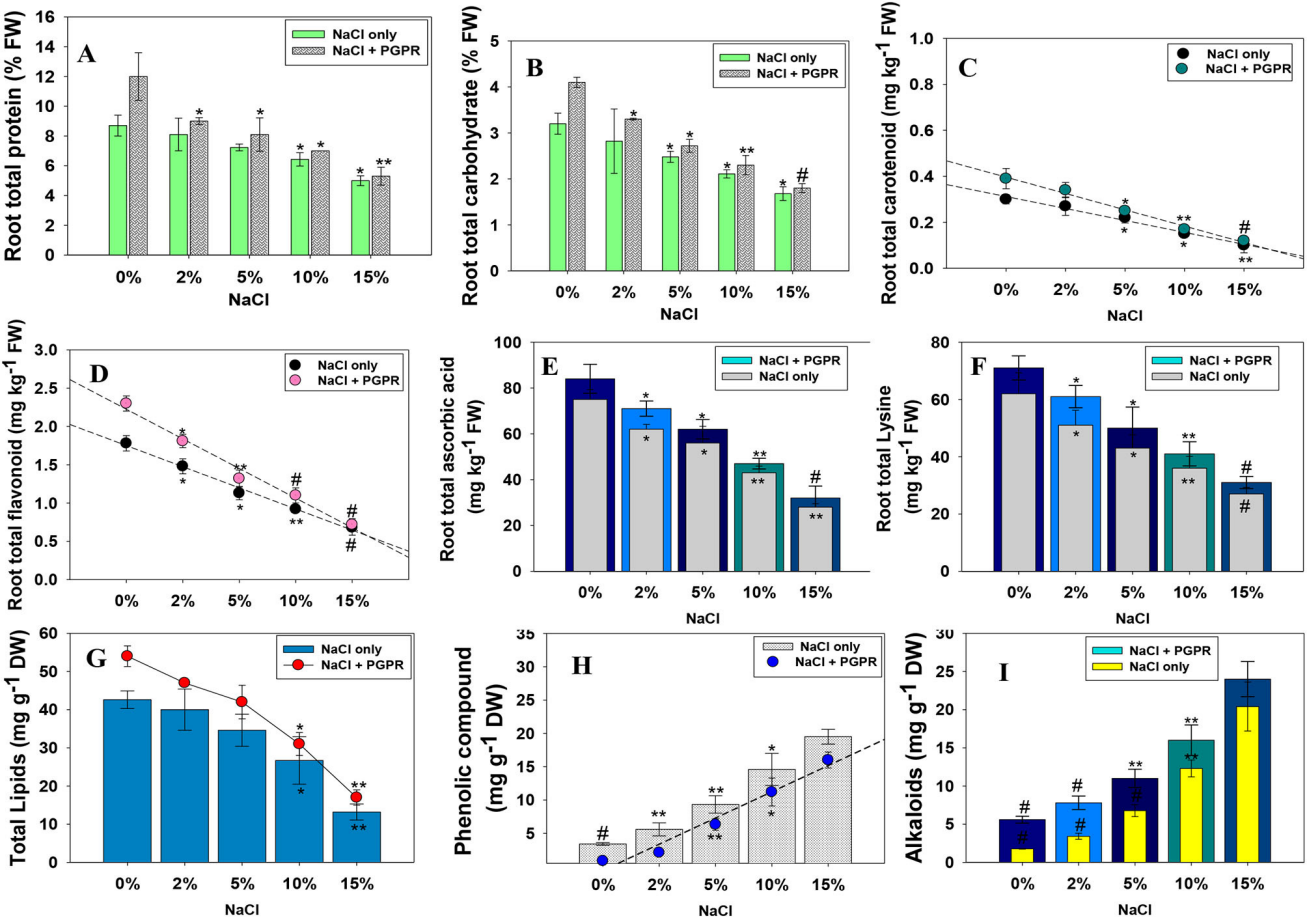
Inoculation impact of halotolerant PGPR strain on root total protein **(A)**, total carbohydrate **(B)**, total carotenoid **(C)**, total flavonoid **(D)**, ascorbic acid **(E)**, lysine content **(F)**, total lipids **(G)**, total phenolics **(H)**, and alkaloids **(I)** extracted from root and foliage tissues of radish plants raised in soils treated with different levels of NaCl. In this figure, bar and line diagrams represent the mean values of three replicates (*n* =3). Corresponding error bars represent standard deviation (SD) of three replicates (SD, *n* = 3). The asterisks *, **, and # denote statistical significance at *p* < 0.05, *p* < 0.005 and *p* < 0.001, respectively, computed by Student's *T*-test.

The increasing concentrations of abiotic stresses, including salts, often resulted in a substantial reduction in total root protein and carbohydrate content. Like other biological features of plants, here, a high NaCl level maximally reduced the protein and carbohydrate content accumulated in root tissues of *R. sativus* (L.). Alternatively, bio-inoculation of salt-tolerating PGPR strain KR-17 relieved the salt stress and significantly increased the protein and carbohydrate. For instance, at 2% NaCl, strain KR-17 maximally increased the amount of protein and carbohydrates in root tissues by 11 and 18%, respectively, over un-inoculated control (Figures 4A,B). This enhancement might be attributed to salt-relieving ability of PGPR strain, which may possibly demonstrate the beneficial impacts on investigated minerals by preserving cell membrane stability/integrity. A similar pattern of NaCl-induced decrease was recorded in carotenoid and flavonoid content accumulated in root tissues, which, however, increased considerably, following the inoculation of salt-tolerating *K*. *radicincitans* (Figures 4C,D).

##### Ascorbic Acid and Lysine Content

Lysine (Lys) is an essential signaling amino acid that stimulates plant development and reactions to environment. In plants, Lys is thought to be involved in various physiological activities, including blooming, formation of seeds, gamete production, and fertilization. Considering the importance of amino acids as building-block material for growth and development of plants, effect of increasing concentrations of NaCl on AsA and Lys content accumulated in root tissues of radish was evaluated. Here, NaCl-induced stress resulted in a substantial reduction in AsA and Lys content. However, radish plants grown from seeds pre-bacterized with KR-17 strain and supplemented with various NaCl regimens showed a substantial improvement in AsA and Lys content. While comparing all treatments, the plants produced from seeds pre-primed with salt-tolerant PGPR and treated with 2% NaCl exhibited a greatest increase of 13% and 16% in AsA (Figure 4E) and Lys content (Figure 4F), respectively.

#### Total Lipids, Phenolics, and Alkaloids

Here, the quantity of total lipids extracted from root tissues of NaCl-treated *R. sativus* (L.) dropped as the concentration of NaCl increased. In contrast, bio-priming of *R. sativus* (L.) seeds with halotolerant PGPR mitigated the salinity stress, and total lipid content was considerably increased. For instance, strain KR-17 maximally improved the total lipids in the presence of 2% NaCl (Figure 4G). The increased lipid synthesis caused by PGPR might be owing to salt-relieving effect on membrane lipids and higher activity of lipid-producing enzymes.

The buildup of phenolic compounds accumulated in leaf tissues of *R. sativus* (L.) was considerably increased by NaCl stress, which was more pronounced at 15% NaCl. This increase might be the result of osmotic stress or an enhanced activity of plant hormones. However, compared to un-inoculated control, the combined application of NaCl and halotolerant KR-17 strain resulted in a proportionate decrease in its level (Figure 4H). Thus, increased accumulation of phenolics under salt stress both with/without PGPR inoculation indicated that induction of secondary metabolism is one of the defense strategies used by plants to cope with under a harsher saline condition. Similarly, NaCl stress has accelerated the accumulation of total alkaloid in radish foliage, which was increased with a rising level of NaCl. However, over un-inoculated control, the KR-17-bacterized plants resulted in a significant decrease in an alkaloids level (Figure 4I). The decrease in alkaloids as a result of PGPR application might be attributed to their salt-tolerating and strong salt-mitigating properties, which protect the plant from damaging effects of reactive oxygen species (ROS) mediated during metabolic processes.

#### Mineral Composition of *R. sativus* (L.) Was Improved by Strain KR-17 and NaCl Stress

**Figure 5:**
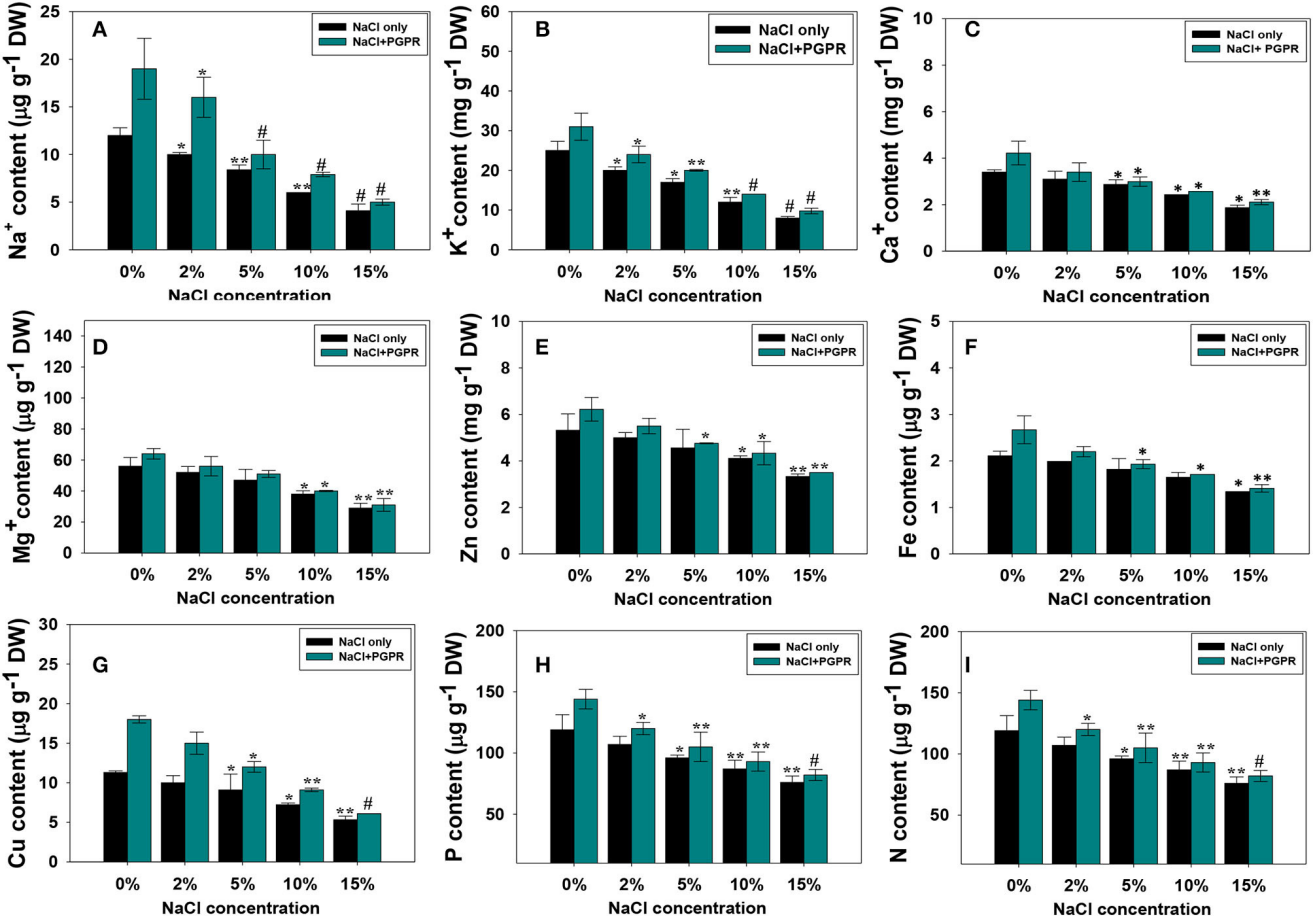
Mineral composition; Na^+^ **(A)**, K^+^ **(B)**, Ca^+^ **(C)**, Mg **(D)**, Zn **(E)**, Fe **(F)**, Cu **(G)**, P **(H)**, and N **(I)** accumulated in root tissues of radish plants raised in soils treated with different levels of NaCl and inoculated with halotolerant PGPR strain. In this figure, bar and line diagrams represent the mean values of three replicates (*n* = 3). Corresponding error bars represent standard deviation (SD) of three replicates (SD, *n* = 3). The asterisks *, **, and # denote statistical significance at *p* < 0.05, *p* < 0.005, and *p* < 0.001, respectively, computed by Student's *T*-test.

In this study, the concentration of minerals in the leaf tissues of *R. sativus* (L.) was considerably reduced with increasing salt stress. Like other plant parameters, the higher salt level (15% NaCl) poses a remarkable decrease in the nutrient uptake related to lower concentrations. The decrease in mineral nutrient absorption under saline circumstances could be due to Na^+^-induced transporter blockage, which results in an ionic imbalance of K^+^, Ca^2+^, and Fe^2+^ as opposed to Na^+^. However, the plants developed from seeds pre-treated with different regimens of NaCl and halotolerant PGPR strains showed a considerable increase in these nutrients. For instance, Na, K, Ca, Mg, Zn, Fe, Cu, P, and N contents were maximally increased by 38, 17, 12, 9, 11, 14, 33, 11, and 15%, respectively, following the soil inoculation of KR-17 strain in the presence of 2% NaCl over non-inoculated but added with a similar rate of salts (Figures 5A–I). The inoculation of halotolerant PGPR strain in *R. sativus* (L.) may help to reduce the negative effects of salt stress. A few mechanisms can explain this phenomenon; bacterial inoculation can inhibit the absorption of Na and Cl ions while positively enhancing the uptake of other plant nutrients, such as Na, K, Ca, and Mg. The N-fixation and P-solubilization may have induced greater total N and P absorption in radish, boosting the development of plants. Higher nutrient absorption by PGPR inoculations resulted in considerably enhanced seedling development. Inorganic ions, particularly Na^+^, might be held in roots by PGPR inoculants, limiting their transport to leaves. By reducing Na^+^ absorption, these bacteria may help to enhance the salt tolerance to crops. By improving water usage efficiency and supplying plants with fixed N, Fe, and soluble P, salt-tolerant PGPR can help plants' roots and thrive in salty environments (Dey et al., 2021). Plant growth and absorption of plant nutrient elements from soil might be boosted in this way by stimulating the development of root system. Therefore, increased root development may contribute to stability of membrane permeability and enhance the synthesis of chlorophyll and RLWC, boosting the growth of *R. sativus* (L.) plants in saline environment. Similarly, very recently, Kusale et al. (2021) have observed that the growth and nutritional content of salinity-stressed maize and plants were considerably increased following the inoculation of halotolerant PGPR. Likewise, in a study, salt-tolerant bacterial strains enhanced the mineral composition of *R. sativus* (L.) plants when applied to soils added with different levels of salts (Yildrim et al., 2008).

#### Halotolerant KR-17 Strain Improved the Stress-Related Parameters (Membrane Damage and Relative Leaf Water Content; RLWC)

**Figure 6:**
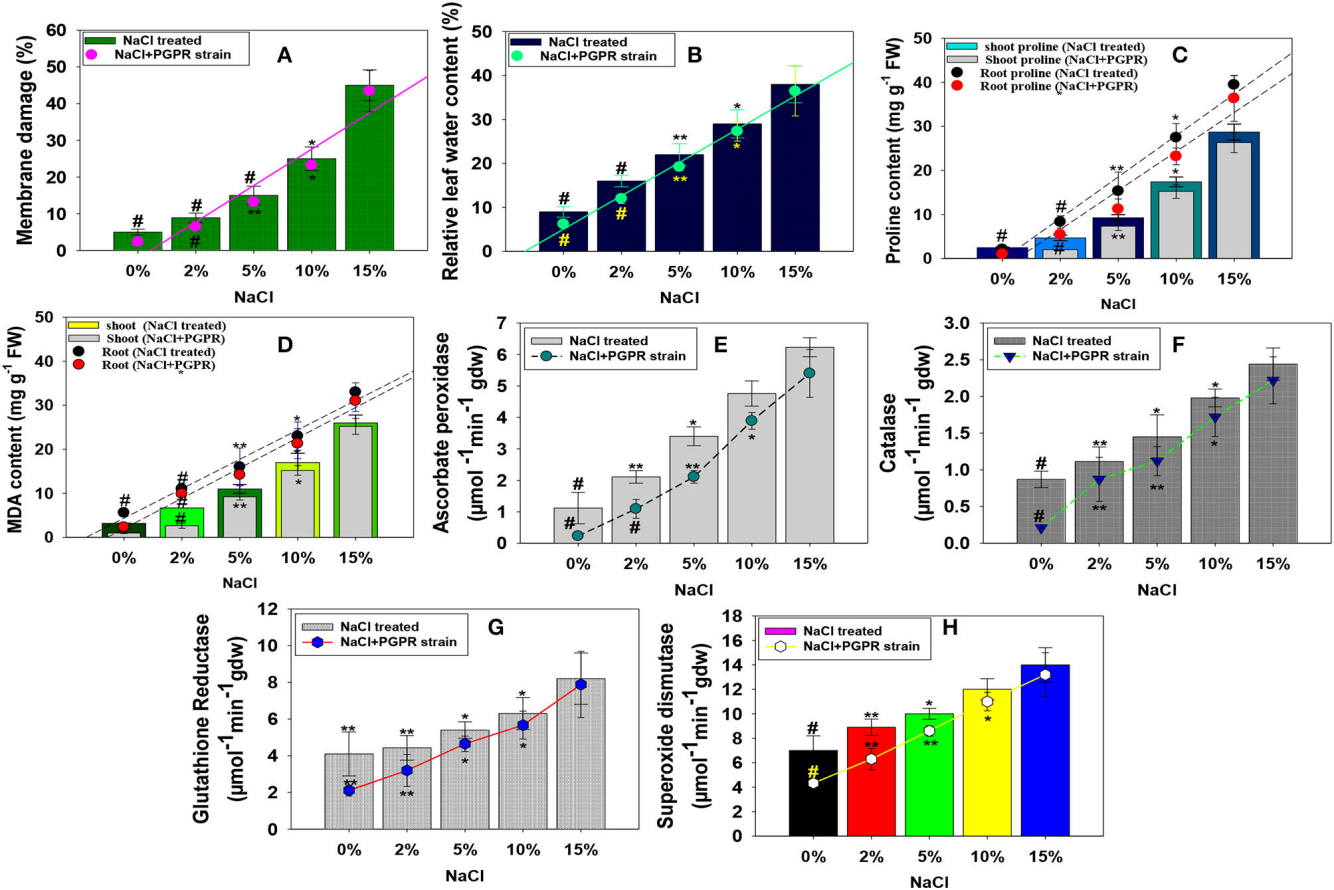
Impact of different levels of NaCl on membrane damage **(A)**, relative leaf water content **(B)**, stressor molecules; proline **(C)**, MDA content **(D)**, and antioxidant enzymes; ascorbate peroxidase **(E)**, catalase **(F)**, glutathione reductase **(G)**, and superoxide dismutase **(H)** of radish plants raised in soils inoculated with halotolerant *K. radicincitans*. In this figure, bar and line diagrams represent the mean values of three replicates (*n* =3). Corresponding error bars represent standard deviation (SD) of three replicates (SD, *n* = 3). The asterisks *, **, and # denote statistical significance at *p* < 0.05, *p* < 0.005, and *p* < 0.001, respectively, computed by Student's *T*-test.

The membrane damage and RLWC in salt-treated and PGPR-inoculated radish plants showed a varied response. As a result, membrane injury and RLWC have been enhanced with a corresponding increase in NaCl concentration. For example, as compared to untreated control, 15% concentration of NaCl increased the membrane injury and RLWC maximally and substantially by 89 and 76%, respectively (Figures 6A,B). However, after soil inoculation, strain KR-17 dramatically reduced the membrane damage potential and RLWC of the plants. The drop in these characteristics might be attributable to the use of halotolerant PGPR strain, which likely lowered the sodium absorption in NaCl-treated *R. sativus*. Similar to our findings, halotolerant PGPR strains greatly reduced the salt stress and improved the membrane damage, relative leaf water content, ionic composition, and production of strawberry plants cultivated in the presence of various levels of salt stress (Karlidag et al., 2010).

#### Bio-Inoculation of *K. radicincitans* KR-17 Affected Stressor Metabolites (Proline and MDA Content)

Salinity stress is typically linked to oxidative damage in terms of reactive oxygen generation (ROS). The ROS comprises of OH–, O^2^-, and H_2_O_2_, as well as free radicals, which damage the plant development and survival (Choudhury et al., 2017). Production of ROS is a key indicator of the damage caused by salt stress. The ROS accumulation in plant tissues causes cellular membrane damage, as well as the oxidation of biological macromolecules (Hasanuzzaman et al., 2020). Stress-inducing proline in plant bodies is seen as physiological and functional activities. Proline, a stressor molecule, shields the cellular organelles and membranes from detrimental effects of increased salt and other inorganic ion concentrations. In many plant species, an increase in free cellular proteins in the presence of different biotic and abiotic stressors serves as a self-protective mechanism (Hussain et al., 2018). Another oxidative stress measure is lipid peroxidation/malondialdehyde (MDA). In this this study, proline and MDA content salt-induced and PGPR-inoculated radish were examined. These stress indicators showed a concentration-dependent increase as the level of NaCl increases. Contrarily, bacterial strain resistant to salt reduced the NaCl-induced oxidative stress. For example, when administered to *R. sativus* (L.), plants in the presence of 2% NaCl concentration, strain KR-17 substantially and maximally decreased the proline and MDA content by 56 and 60%, respectively (Figures 6C,D). Likewise, Islam et al. (2016) in a parallel investigation also observed that plant growth-promoting halotolerant strain *Bacillus cereus* strain Pb-25 alleviated the NaCl-induced toxicity and improved proline and MDA content in *V. radiata* L. (greengram) *via* the upregulation of antioxidant defense enzymatic activities.

#### *K. radicincitans* Modulated the Antioxidant Defense Enzymes of NaCl-Treated Radish

Plants develop antioxidant mechanisms to prevent the startling impacts to alleviate the oxidative stress. Antioxidant enzymes like ascorbate peroxidase (APX), superoxide dismutase (SOD), catalase (CAT), and glutathione reductase (GR) are widely known and common enzymes that are produced by various cellular organelles, such as mitochondria and chloroplasts, which play an essential part in the protection of biological systems (García-Caparrós et al., 2021). A reactive oxygen species (ROS) buildup activates SOD, which results in production of hydrogen peroxide (H_2_O_2_,), a harmful signal molecule for oxidative stress (Liu et al., 2018). The buildup of H_2_O_2_ enhances the activities of POD, CAT, APO, and GPO in order to reduce its concentration by converting it to O_2_ and H_2_O. In the current study, it was discovered that, when concentration of NaCl increased from low (2% NaCl) to high (15% NaCl), the level of antioxidant enzymes in leaf tissues of *R. sativus* (L.) increased. Among the concentrations tested, 15% NaCl had the most negative impact and elevated the antioxidant enzymes. For example, at 15% NaCl concentration, APX, CAT, GR, and SOD enzymatic activities accumulated in leaf tissues of *R. sativus* (L.) were increased significantly (*p* ≤0.005) by 82, 64, 51, and 50%, respectively, related to the untreated control (Figures 6E–H). However, by ameliorating the negative effects of salinity stress, halotolerant KR-17 strain decreased the amount of antioxidant enzymes. For example, in the presence of a 2% NaCl range, KR-17 dramatically lowered the APX, CAT, GR, and SOD levels by 52, 21, 27, and 30%, respectively, compared to un-inoculated but treated with a similar rate of salts (Figures 6E–H). The reduced antioxidant enzyme expression in KR-17-treated *R. sativus* (L.) plants grown in soils supplemented with varying levels of NaCl might be linked to decreased Na^+^ absorption and, as a result, lesser oxidative damage. Similar to this, biofilm-forming halotolerant PGPR strains *Bacillus licheniformis* and *Pseudomonas plecoglossicida* enhanced growth of sunflower plants by increasing the salt tolerance and *via* stimulating antioxidant enzymes (Yasmeen et al., 2020).

#### Biofilm Formation (*in vitro*) and Root Colonization by Halotolerant PGPR (*in vivo*)

**Figure 7:**
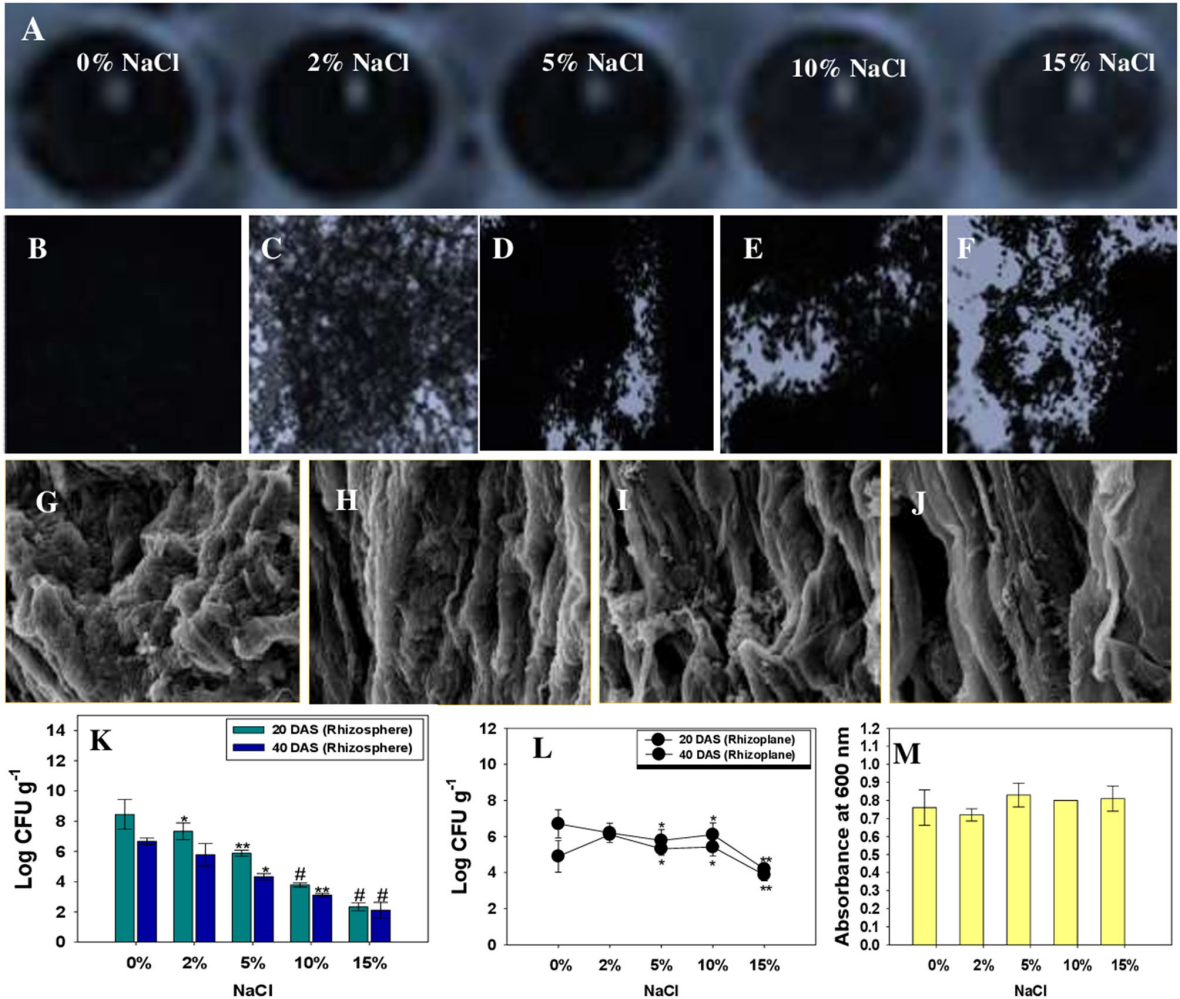
*In vitro* biofilm formation by *K. radicincitans* co-cultivated with different levels of NaCl in polystyrene wells **(A)** and on A glass surface: control **(B)**, 2% NaCl **(C)**, 5% NaCl **(D)**, 10% NaCl **(E)**, and 15% NaCl **(F)**. Scanning electron micrographic images of *R. sativus* roots treated with different levels of NaCl and colonized by *K. radicincitans* **(G–K)**. **(L,M)** represent Log CFU of *K. radicincitans* g^−1^ measured from radish rhizosphere (CFU g^−1^) and rhizoplane (CFU g^−1^), whereas **(N)** depicts the mean ± S.D. absorbance of *in vitro* biofilm by a crystal violet assay. The asterisks *, **, and # denote statistical significance at *p* < 0.05, *p* < 0.005, and *p* < 0.001, respectively.

The ability of beneficial microorganisms to colonize the plant roots is critical to rhizosphere plant-microbe interaction, which aids plant development and safeguard the crops against several abiotic and biotic elements (Gupta et al., 2020; Santoyo et al., 2021). Under adverse environmental condition, adhesion of beneficial microbial cells like halotolerant *K. radicincitans* to the plant surface, known as the biofilm, is a critical step for development and fortification of agricultural crops against harmful factors, including salinity. In natural settings, bacteria prefer to live in biofilms rather than planktonic cells (Nievas et al., 2021). The root colonization of *R. sativus* (L.) by KR-17 cells co-cultivated with NaCl, on the other hand, is a mystery. In this work, we investigated the biofilm formation by *K. radicincitans* on a glass surface (*in vitro*) (Figure 7A) and on the roots of *R. sativus* (L.) plants grown in soil amended with salt. Under *in vitro*, and on both polystyrene wells and glass cover-slips, KR-17 strain was proved to be a positive biofilm producer (Figures 7B–F). There were no significant differences in the biofilm formation of bacterial strain observed after exposure to 15% NaCl. There was just a little difference in mean absorbance (λ_600_ nm). A typical biofilm development by *K. radicincitans* was also visible on the control glass cover slip. Plant beneficial bacteria behave differently in the rhizosphere to plant secretions known as root exudates. Root secretions function as crucial indicators for reproduction and colonization of bacteria as biofilms on the rhizoplane (Zhang et al., 2014). After 45 days of plant growth, SEM micrographs of un-treated and NaCl-treated groups revealed the colonization of the rhizoplane region by *K. radicincitans* (Figures 7G–K). This might be related to bacterial chemotactic reaction to root exudates of radish, as well as the combined involvement of bacterial extracellular proteins, cell wall polysaccharides, and EPS in promoting the productive root surface adhesion. The effective establishment of *K. radicincitans* and its growth even in salt-challenged soil was further confirmed by colony-forming unit (CFU) counts of rhizosphere soil obtained from unexposed and NaCl-exposed soils and rhizoplane (Figures 7L–N). Similarly, salt-tolerant PGPR strains *Bacillusvelezensis, B. altitudinis*, and *B. safensis* colonized the *Zea mays* (L.) roots inoculated separately or in consortia and developed a thin biofilm on the root system under saline condition (Singh et al., 2021).

## Conclusion

The present finding suggests that inoculating *R. sativus* (L.) with salinity-alleviating (i.e., halotolerant) PGPR isolate, expressing a variety of plant growth-promoting characteristics, might improve growth, redox potential, and ion homeostasis, therefore facilitating the development of a vegetable crop. Under harsh saline conditions, inoculated *K. radicincitans*, comprising the potential ability of biofilm formation, have shown a substantial increase in the growth promotion of *R. sativus* (L.). A key contributor to plant development and salt stress tolerance are integral growth features of chosen halotolerant PGP bacteria, comprising the activity of P-solubilization, indole-3-acetic acid, and ACC deaminase synthesis. Inoculating salinity-exposed *R. sativus* (L.) plants with *K. radicincitans* KR-17 improved the dry biomass, chlorophyll synthesis, mineral composition, redox status, reduced stressor metabolites, and antioxidant defense enzymes. Overall, these results confirm the future synchronization of functions between the two symbionts (*R. sativus* L. and salt-tolerant *K. radicincitans* KR-17). As a result, it may be inferred that biofilm-forming and halotolerant bacterium *K. radicincitans* carrying multifarious PGP properties might be produced as inoculants to help vegetable crops cultivated in salty soils cope with salinity stress. Furthermore, molecular signals and mechanisms that drive beneficial plant-microbe interactions are still little understood, and even less is known about the link between growth-regulating molecules in PGPR-primed plants and their overall response to salinity stress. The use of a systems biology approach to untangle and comprehend the complexities of plant-microbe interactions under NaCl-stress opens up new avenues for using soil beneficial microbes as long-term crop enhancement agents. A complete and multi-omics-based investigation is required to examine physiological responses with proper validation and testing of hypothesis *via in vitro* and *in vivo* tests, and this should be the key next step.

## Summary

Salinity stress among abiotic stress is one of the major variables affecting agricultural areas and reducing crop productivity. Among leafy vegetables, radish is an important vegetable crop (containing a high amount of vitamins “A” and “C” as well as minerals like “sulfur”) consumed by people. We employed a NaCl-tolerant bacterium *Kosakonia radicincitans* KR-17 that produces high amounts of essential PGP metabolites and colonizes the roots of plants to tackle the problem of salinity-affected radish. We find that strain KR-17 has considerable potential to reduce salt stress after conducting several *in vitro* and *situ* (on-field) tests. The bacterial activity, which inhibited the NaCl stress, increased the number of agriculturally relevant biometric parameters in radish. In conclusion, strain KR-17 can be used to relieve salinity stress by increasing the biological properties of radish under salt stress.

## Data Availability Statement

The datasets presented in this study can be found in online repositories. The names of the repository/repositories and accession number(s) can be found in the article/Supplementary Material.

## Author Contributions

FA-K: conceptualization, data curation, formal analysis, investigation, methodology, resources, software, validation, visualization, writing—original draft, and writing—review and editing. MS: data curation, formal analysis, investigation, methodology, resources, software, validation, visualization, writing—original draft, and writing—review and editing. MD: formal analysis, writing—review and editing, methodology, resources, and software. AA: formal analysis, writing—review and editing, methodology, software, funding, and resources. AM: methodology, resources, and funding. SA: writing—review and editing, methodology, and resources. All authors contributed to the article and approved the submitted version.

## Conflict of Interest

The authors declare that the research was conducted in the absence of any commercial or financial relationships that could be construed as a potential conflict of interest.

## Publisher's Note

All claims expressed in this article are solely those of the authors and do not necessarily represent those of their affiliated organizations, or those of the publisher, the editors and the reviewers. Any product that may be evaluated in this article, or claim that may be made by its manufacturer, is not guaranteed or endorsed by the publisher.
