# Supplementary material for: PGPR Kosakonia Radicincitans KR-17 Increases the Salt Tolerance of Radish by Regulating Ion-Homeostasis, Photosynthetic Molecules, Redox Potential, and Stressor Metabolites
Source: Front Plant Sci. 2022 Aug 1;13:919696. doi: 10.3389/fpls.2022.919696 (PMC9376370; doi:10.3389/fpls.2022.919696)
Supplement: Supplementary file 1 [file Data_Sheet_1.docx]

**Electronic Supporting Information**

**PGPR *Kosakonia radicincitans* KR-17 increases the salt tolerance of Radish by regulating ion-homeostasis, photosynthetic molecules, redox potential and stressor metabolites**

*Mohammad Shahid*^a^*, Fatimah S. Al-Khattaf*^b^*, Mohammad Danish*^c^*, Ashraf Atef Hatamleh*^b^*, Abdullah Mohamed*^d^*, Sajad Ali*^e^*

*^a^Department of Agricultural Microbiology, Faculty of Agricultural Sciences, Aligarh Muslim University, Aligarh-202002; Uttar Pradesh, India*

*^b^Department of Botany and Microbiology, College of Sciences, King Saud University, P.O. Box 2455, Riyadh 11451, Saudi Arabia*

*^c^Section of Plant Pathology and Nematology, Department of Botany, Aligarh Muslim University, Aligarh-202002; Uttar Pradesh, India*

*^d^Research Centre, Future University in Egypt, New Cairo, 11745, Egypt*

*^e^Department of Biotechnology, Yeungnam University, Gyeongsan 38541, South Korea*

**Correspondence to*:

Dr. Mohammad Shahid

Department of Agricultural Microbiology,

Faculty of Agricultural Sciences,

Aligarh Muslim University, Aligarh, India

+91-08090939511

**E-mail:** [shahidfaiz5@gmail.com](mailto:shahidfaiz5@gmail.com)

**Supplementary Methods**

***2.3 Identification of rhizobacterial strains using 16SrRNA gene sequencing***

The total genomic DNA was extracted by the method as previously described by Yadav et al. (2011). The forward primer pA (5’AGA GTT TGA TCC TGG CTC AG3’) and reverse primer pH (5’AAG GAG GTG ATC CAG CCG CA3’) (Solanki et al., 2012) were used to amplify the 16S rRNA gene from genomic DNA. The total volume of reaction mixture was 100 µL containing 50–80 ng of template DNA, 10X reaction buffer, 2.5 mM dNTPs, 20 pM of each primer and one-unit Taq DNA polymerase (Bangalore Genei, India), and reactions were performed on G-storm thermocycler (G-STORM, UK). The amplification conditions were as follows: initial denaturation at 94 °C for 5 min, followed by 35 cycles of denaturation at 94 °C for 40 s, annealing at 52 °C for 45 s, and elongation at 72 °C for 1 min 30s. At the end of 35 cycles, the final extension step was at 72 °C for 8 min. The amplified product was resolved by electrophoresis in 1.2% agarose gel in 1X TAE buffer. Gels were stained with ethidium bromide (EtBr 10 mg mL^-1^) and visualized on gel documentation system (BIO-RAD, USA). Strong and clear bands were scored for similarity and clustering analysis using the software, NTSYS-2.02e package (Numerical taxonomy analysis program package, Exeter software, USA). The purified 16S rRNA PCR products were send to Macrogen, Seol, south Korea for 16S rRNA sequencing. The 16S rRNA amplicons were sequenced from both ends and consensus sequence was generated. The partial 16S rRNA gene sequences were compared with those available in the databases (http://www.ncbi.nlm.nih.gov/BLAST/) and identification to the species level was determined on the basis of sequence similarity of >97% with the closest relative in the GenBank. The phylogenetic tree was constructed on the aligned datasets using the neighbor-joining method implemented in the program MEGA 7.0 software. Bootstrap analysis was performed on 1,000 random samples taken from the multiple alignments.

**S2.4 *Bioassays for growth regulating substances under NaCl stress***

**S2.4a *Indole-3-acetic acid (IAA), siderophore and ACC deaminase enzyme***

The plant hormone, indole-3-acetic acid (IAA) produced by *K*. *radicincitans* strain KR-17 was quantitatively assayed by the modified method of Bric et al. (1991). Here, bacterial strain was cultured in Luria Bertani (LB) broth (gL^-1^: tryptone 10; yeast extract 5; NaCl 10 and pH 7.5). A- 100 mL of LB broth containing fixed concentration of tryptophan (100 mg mL^-1^) was treated with 0, 2, 5, 7, 10 and 15% NaCl concentrations. The salt containing LB broth was then inoculated with 100 μL culture (10^8^ cells mL^-1^) of KR-17 strain and incubated at 28±2 °C for four days with shaking at 120 r/min. Following complete incubation, culture (5.0 mL) was centrifuged (8000 r/min) for 10 min. and two mL supernatant was added with 100 μL of orthophosphoric acid (H_3_PO_4_) and four mL Salkowsky reagent (2% 0.5 M FeCl_3_ prepared in 35% per-chloric acid) and incubated for one hour at 28±2°C in dark for colour development. The absorbance of pink color developed during reaction was measured at 530 nm. The quantity of indole acetic acid was calibrated using pure IAA as a standard.

**S2.4b *Bioassay for siderophore production***

The isolate was spot inoculated on NaCl supplemented universal chrome azurol S (CAS) agar plates followed by incubation at 28±2 °C for detection of orange color halo around the bacterial colonies. Furthermore, the production of siderophore was quantitatively assessed by growing the bacterial strain in NaCl amended iron (Fe) free succinate liquid medium as suggested by Barbhaiya and Rao (1985). The estimation of siderophore was done according to universal chrome azurol liquid assay (Schwyn and Neilands 1987). Siderophore units were calculated as follows:

% Siderophore unit =$\frac{\lambda of reference \left( \mathrm{Ar} \right)-\lambda of test (As)}{\lambda of reference (Ar)}\times100$

Where, Ar = Absorbance of uninoculated media and CAS solution

As = Absorbance of test sample i.e. culture supernatant and CAS solution

Furthermore, phenolate siderophore salicylic acid (SA) and 2,3-DHBA was determined (Alexander and Zuberer, 1991)

**S2.4c *Bioassay for ACC Deaminase Activity***

The plant growth modulating enzyme ACC deaminase (EC 4.1.99.4) secreted by *K*. *radicincitans* was qualitatively detected by spot inoculation method using Dworkin and Foster (DF) salts minimal medium (Dworkin and Foster, 1958) containing 3.0 mM ACC as nitrogen (N) source. Plates containing DF medium without ACC and with (NH_4_)_2_SO_4_ (0.2% w/v) served as negative and positive control, respectively. Plates maintained at 28±2°C for 72 h were examined each day for bacterial growth. *Mesorhizobium* LMS-1 containing pRKACC plasmid (Shah et al., 1998) was used as a positive control. The ACC deaminase secreted by bacterial strain grown in the presence of different levels of NaCl was extracted as suggested by Honma and Shimomura (1978) and Penrose and Glick (2003). The quantity of α-ketobutyrate generated due to ACC deaminase activity was measured spectrophotometrically against a standard curve of α-ketobutyrate. The activity of ACC deaminase was presented as the quantity of α-ketobutyrate released/mg of protein/h. All individual experiments were conducted 3 times on different intervals.

**S2.5 *Biofilm development*** ***assessment***

Biofilm development on microtiter plate (96-well plate) in the presence and absence of NaCl was assayed adopting the standard procedure described by (O’Toole 2011) using 1% crystal violet (CV) to stain biofilms formed in microtiter plate. Biofilm bounded dye was extracted in 75% ethanol, and absorbance was measured at 595 nm in microtiter plate reader (Thermo Scientific Multiskan EX, UK) to examine the concentration of dye retains by the biofilm and compared with control (without salt). Each treatment was replicated in five independent wells for biofilm quantification, and experiment was repeated three times.

***S2.5.1 assessment of alginate production***

Alginate produced by KR-17 strain was quantified. For assessment, cells were grown in liquid medium added with different concentrations of NaCl. Supernatant obtained after centrifugation and filtration was subjected to deacetylated alginate isolation by adding the isopropanol in equal volume and stored at room temperature for 24 h. Precipitate was collected by centrifugation for 10 min at 10,000 rpm followed by washing with graded concentrations of ethanol (70%). The washed and dried pellet was suspended in 1.0 mL of DW. Further, 100 μL of suspension was kept in fresh tubes and made the volume up to 1.0 mL by adding Milli-Q water for the quantification. Freshly prepared 1.0 mL of borate sulfuric acid solution (10 mM) was supplemented after the addition of fresh carbazole reagent (30 μL) and then mixed properly. The solution mixture was left at room temperature for 15 min and read the absorbance at 500 nm against reagent blank. The quantitative extraction of alginate was expressed in terms of μg/mg wet biomass (Wozniak et al. 2003).

**S2.6 *Crop-Based Experiments***

**S2.6.1 *Plant culture, NaCl treatment and inoculation of K. radicincitans***

Healthy seeds of radish (*R. sativus* L.) were surface sterilized using 2% sodium hypochlorite (NaOCl), washed thoroughly, cleaned and desiccated at room temperature. Stock solution of sodium chloride (NaCl) was prepared and 0 (untreated control), 2%, 5%, 10% and 15% NaCl solutions were applied to moist soils at least 1 day before sowing seeds. The physiochemical properties of the soil are shown in Supplementary Table S1. The soil was placed into 20 × 24 cm clay pots with approximately 5 kg soil per pot. The disinfected radish seeds were coated with cultures of Kosakonia radicincitans KR-17 (grown for 48 h) by immersing them in liquid culture medium for 2 h using 10% gum Arabic as an adhesive to achieve 1 × 10^8^ cells seed^−1^. Un-inoculated sterilized seeds submerged in sterile water were used as control. Seeds (n = 10) were sown in respective earthen pots containing 5 kg of soil. Sowing was carried out and germination was recorded 7 days after sowing (DAS). Two controls were run in parallel; one was uninoculated and untreated (without bacteria and without NaCl) and the second was inoculated (bacteria but no salts). Pots without NaCl treatment but bacterized (i.e., coated with PGPR strain) radish seeds also served as a control treatment for comparison. Exposure at each NaCl concentration was replicated thrice and pots were arranged in a completely randomized block design. After germination, seedlings were thinned and two uniform healthy seedlings of radish were maintained in each pot, 15 days after emergence (DAE). Pots were kept in an open field condition (9 h light/15 h dark cycle) and watered regularly using tap water. The crop experiments were carried out for 2 years to achieve consistency and reproducibility in results.

In order to better understand, the plan for experimental treatment is as below:

T1= Control (Untreated control)

T2= Inoculated control (inoculated the bacteria)

T3= treated with 2% NaCl

T4= treated with 5% NaCl

T5= treated with 10% NaCl

T6= treated with 15% NaCl

T7= 2% NaCl + bacterial strain KR-17

T8= 5% NaCl + bacterial strain KR-17

T9= 10% NaCl + bacterial strain KR-17

T10= 15% NaCl + bacterial strain KR-17

**S2.6.5 *Estimation of root ascorbic acid and lysine content***

The content of Ascorbic acid (AsA) in freshly removed root tissues of PGPR inoculated and NaCl-treated *R. sativus* plants were estimated (Mukherjee and Choudhuri, 1983). The fresh plant material (5.0 g) was homogenized in 20% trichloro acetic acid (TCA). After filtration and centrifugation (10000 × *g*), one mL of the supernatant was reacted with 2 mL of 2% dinitrophenyl hydrazine solution (in acidic medium). Then one drop of ethanolic 10% thiourea solution was added to the mixture. The mixture was boiled for 20 min in a water bath. After cooling, 5.0 mL of 80% H_2_SO_4_ (v/v) were added to the mixture. The absorbance was read at 530 nm. The amount of AsA in the extracted leaf samples was worked out from a standard curve prepared using varying AsA standards.

**S2.6.6 *Determination of mineral composition in root tissues of R. sativus***

The uptake of mineral content in root tissues of NaCl treated and bio-inoculated *R. sativus* was determined. For the estimation, 0.1 g of root samples were oven-dried following acid digestion. Mineral content like sodium (Na), calcium (Ca), potassium (K), magnesium (Mg), iron (Fe), zinc (Zn), copper (Cu), phosphorous (P) and nitrogen (N) etc. were determined using spectrophotometrically and by mean of atomic absorption spectrophotometer (AAS). For the assay, plant samples were oven-dried and from the dried material, 0.1 g of root samples was taken in digestion flasks. A 5.0 mL of conc. H_2_SO_4_ (sulphuric acid) was carefully added to each flask (Wolf, 1982) and incubated overnight at room temperature. Afterwards, 0.5 mL of 35% hydrogen peroxide (H_2_O_2_) was added to it and the flasks were then placed over a hot plate at 100 ^0^C that was gradually raised to 380 ^0^C until no fumes were produced. The digestion flasks were then removed from the hot plate and allowed to cool. After addition of 0.5 mL of H_2_O_2_, flasks were again placed on a hot plate (380 ^0^C). This step was repeated until the digestion mixture became transparent. The mixtures were diluted up to 50 mL in volumetric flasks, filtered and stored at 4 ^0^C till further elemental analysis. The content of Ca, K and Na were estimated using flame photometer. Whereas, Zn, Mg, Fe and Cu were assessed by AAS using flame spectrophotometry. The phosphorus (P) content of the digested plant material was estimated spectrophotometrically using Barton's reagent (Ekholm, 2007).

**S2.6.8.2 *Estimation of free proline in R. sativus organs***

For the assay, one-gram of fresh plant organ was homogenized with 5.0 mL of 3% (w/v) aqueous sulfosalicylic acid (C_7_H_6_O_6_S). The resulting homogenate was filtered through Whatman No.2 filter paper. The resulting cell extract was then centrifuged (at 10000 ×*g* for 20 min) to remove the cell debris. The cell filtrate (2.0 mL) with free proline was treated with 2.0 mL of acid ninhydrin and glacial acetic acid (2.0 mL) at 80 °C for one h. The mixture was heated in boiling water bath for one hour. The reaction was terminated by placing the tubes in ice bath. A- 4.0 mL of toluene was added to the reaction mixture and stirred well for 20-30 seconds. Colored complex was extracted in toluene and the toluene layer was separated. The red color intensity was measured at 520 nm. A series of standard with pure proline was run in a similar way by dissolving proline in 3% C_7_H_6_O_6_S and a standard curve was prepared (Ábrahám et al., 2010). Amount of free proline in the test sample was determined from the standard curve. Proline content on fresh weight basis (μ moles per gram of fresh weight of tissue) was expressed as:

$$Proline content =\frac{\mu g proline/ mL\times mL oftoluene}{115.5}\times\frac{5}{gofsample}$$

**S2.6.8 *Extraction and determination of antioxidant enzymes***

For antioxidant enzyme activity, salt treated and PGPR inoculated radish plants were detached from soil system and foliage were crushed in 4.0 mL of enzyme extraction buffer [(50 mM phosphate buffer (pH=7.8)] containing 1 mM EDTA and 2% (w/v) polyvinylpyrrolidone (PVP). All enzyme assays were performed three times with three replicates of each assay.

***Ascorbate peroxidase (APX)***

The APX activity was determined following the method as described by Shi et al. (2005).

***Catalase (CAT) activity***

CAT activity was measured according to the method of Patterson (1984), with minor modifications. The reaction mixture (3.0 mL) consisted of 100 mM phosphate buffer (pH=7.0), 0.1 mM EDTA and 20 mM hydrogen peroxide (H_2_O_2_). The reaction was initiated by adding 100 µL of enzyme extract. The decrease in H_2_O_2_ was monitored at 240 nm and CAT activity was quantified by using the molar extinction coefficient of H_2_O_2_ (36 mol^-1^cm^-1^).

***Superoxide dismutase* (SOD)**

Activity of SOD was assessed based on ability to inhibit photochemical reduction of nitro-blue tetrazolium (NBT) (Chakrabarti and Patra, 2013). The 3.0 mL of reaction mixture comprising of 13 mM methionine (0.1 mL), 50 mM NaHCO_3_ (0.1 mL), 25 mM NBT (0.1 mL), 0.1 mM EDTA (0.1 mL), 50 mM buffer (2.3 mL) and 0.2 mL of enzyme extract. To this, 2 mM riboflavin was added as end product. This reaction mixture was exposed to 15 W ﬂuorescent tubes for 10 min following incubation for 20 min (in dark). Absorbance was measured at 560 nm.

***Glutathione reductase (GR)***

For GR activity, 0.2 mL of enzyme extract was added to 2.8 mL reaction mix. (H_2_O, buffer, 30 mM GSSG, 0.8 mM NADPH and 1 % BSA) and the absorbance was measured at 340 nm for 3 min (Foyer et al., 1991). The enzyme activity was expressed in µmol ascorbate oxidized min^-1^ g^-1^ dw.

**References**

Ábrahám, E., Hourton-Cabassa, C., Erdei, L. and Szabados, L., 2010. Methods for determination of proline in plants. In: *Plant Stress Tolerance*. Humana Press. 317-331. <https://doi.org/10.1007/978-1-60761-702-0_20>

Alexander, D. B. and Zuberer, D. A., 1991. Use of chrome azurol S reagents to evaluate siderophore production by rhizosphere bacteria. *Biol. Fert. Soils*. 12, 39-45.  <https://doi.org/10.1007/BF00369386>

Barbhaiya, H. B. and Rao, K. K., 1985. Production of pyoverdine, the fluorescent pigment of *Pseudomonas aeruginosa* PAO1. *FEMS Microbiol. Lett*. 27, 233-235. <https://doi.org/10.1111/j.1574-6968.1985.tb00673.x>

Bric, J. M., Bostock, R. M. and Silverstone, S. E., 1991. Rapid *in situ* assay for indole acetic acid production by bacteria immobilized on a nitrocellulose membrane. *Appl. Environ. Microbiol*. 57, 35-538. <https://doi.org/10.1128/aem.57.2.535-538.1991>

Chakrabarti, S. and Patra, P. K., 2013. Effect of fluoride on superoxide dismutase activity in four common crop plants. *Fluoride*, 46, 59-62.

Dworkin, M. and Foster, J. W., 1958. Experiments with some microorganisms which utilize ethane and hydrogen*. J. Bacterial*. 75, 92-603.

Ekholm, P., Reinivuo, H., Mattila, P., Pakkala, H., Koponen, J., Happonen, A., Hellström, J. and Ovaskainen, M.L., 2007. Changes in the mineral and trace element contents of cereals, fruits and vegetables in Finland. *J. Food Com. Analysis*. 20, 487-495. <https://doi.org/10.1016/j.jfca.2007.02.007>

Foyer, C., Lelandais, M., Galap, C. and Kunert, K.J., 1991. Effects of elevated cytosolic glutathione reductase activity on the cellular glutathione pool and photosynthesis in leaves under normal and stress conditions. *Plant Physiol*. 97, 863-872. <https://doi.org/10.1104/pp.97.3.863>

Honma, M. and Shimomura, T., 1978. Metabolism of 1-aminocyclopropane-1-carboxylic acid. *Agric. Biol. Chem*. 42, 1825-1831.  <https://doi.org/10.1080/00021369.1978.10863261>

Mukherjee, S. P. and Choudhuri, M.A., 1983. Implications of water stress‐induced changes in the levels of endogenous ascorbic acid and hydrogen peroxide in *Vigna* seedlings. *Physiol. Plant*. 58, 166-170.  <https://doi.org/10.1111/j.1399-3054.1983.tb04162.x>

O'Toole, G. A., 2011. Microtiter dish biofilm formation assay. *J. Visual. Exp.* 47.  doi: [10.3791/2437](https://dx.doi.org/10.3791/2437)

Patterson, B. D., Payne, L. A., Chen, Y. Z. and Graham, D., 1984. An inhibitor of catalase induced by cold in chilling-sensitive plants. *Plant Physiol.* 76, 1014-1018. <https://doi.org/10.1104/pp.76.4.1014>

Penrose, D. M. and Glick, B. R., 2003. Methods for isolating and characterizing ACC deaminase‐containing plant growth‐promoting rhizobacteria. *Physiol. Plant*. 118, 10-15. <https://doi.org/10.1034/j.1399-3054.2003.00086.x>

Schwyn, B. and Neilands, J.B., 1987. Universal chemical assay for the detection and determination of siderophores. *Anal Biochem*. 160, 47-56. <https://doi.org/10.1016/0003-2697(87)90612-9>

Shi, Q., Bao, Z., Zhu, Z., He, Y., Qian, Q. and Yu, J., 2005. Silicon-mediated alleviation of Mn toxicity in *Cucumis sativus* in relation to activities of superoxide dismutase and ascorbate peroxidase. *Phytochem*. 66, 1551-1559. <https://doi.org/10.1016/j.phytochem.2005.05.006>

Wolf, B., 1982. A comprehensive system of leaf analyses and its use for diagnosing crop nutrient status. Comm. *Soil Sci. Plant Anal*. 13, 1035-1059. <https://doi.org/10.1080/00103628209367332>

Wozniak, D.J., Wyckoff, T.J., Starkey, M., Keyser, R., Azadi, P., O'Toole, G.A. and Parsek, M.R., 2003. Alginate is not a significant component of the extracellular polysaccharide matrix of PA14 and PAO1 *Pseudomonas aeruginosa* biofilms. *Proc. Nat. Acad. Sci*. 100, 7907-7912. <https://doi.org/10.1073/pnas.1231792100>

**Supplementary Tables**

**Table S1:** physico-chemical properties of experimental soil used in the study

| **Parameters** | **Values** |
| --- | --- |
| Organic content (g/kg) | 5.4 |
| Total N (g/kg) | 0.76 |
| Total P (g/kg) | 13.2 |
| Ca (g/kg) | 0.09 |
| Mg (g/kg) | 0.07 |
| Na (g/kg) | 0.12 |
| K (g/kg) | 0.17 |
| Total salt (g/kg) | 3.11 |
| Water holding capacity (ml/g) | 0.37 |
| Cation exchange capacity (cmol/kg) | 8.23 |
| Anion exchange capacity (cmol/kg) | 4.11 |

**Table S2:** Tolerance of NaCl to PGPR strains isolated from rhizosphere soils

| **S. No** | **PGPR strains** | **Tolerance to NaCl (%)** |
| --- | --- | --- |
| 1 | KR-1 | 3% |
| 2 | KR-2 | 5% |
| 3 | KR-3 | 7% |
| 4 | KR-4 | 2% |
| 5 | KR-5 | 8% |
| 6 | KR-6 | 10% |
| 7 | KR-7 | 12% |
| 8 | KR-8 | 15% |
| 9 | KR-9 | 6% |
| 10 | KR-10 | 8% |
| 11 | KR-11 | 10% |
| 12 | KR-12 | 10% |
| 13 | KR-13 | 6% |
| 14 | KR-14 | 8% |
| 15 | KR-15 | 4% |
| 16 | KR-16 | 8% |
| 17 | KR-17 | 18% |
| 18 | KR-18 | 10% |
| 19 | KR-19 | 10% |
| 20 | KR-20 | 10% |

**Table S3:** Microbiological, cultural and biochemical features of *K. radicincitans*

| **Characteristics** | **Strain KR-17** |
| --- | --- |
| *Morphology* | Irregular margin and mucoid colony |
| Gram reaction | -ve |
| Shape  Pigmentation | Short Rods  No |
| Optimum temp. for growth | 30 ^0^C |
| Optimum pH | 7.0 |
| *Biochemical reactions* |  |
| Citrate utilization | + |
| Indole | - |
| Methyl red | - |
| Nitrate reduction | + |
| Hydrogen sulphide production | - |
| Oxidase | - |
| Catalase | + |
| Voges Proskaur | - |
| *Carbohydrate utilization* |  |
| Dextrose | + |
| Lactose | + |
| Mannitol | - |
| Sucrose | + |
| *Hydrolysis* |  |
| Starch | + |
| Gelatin | - |
| *Enzymatic profile* |  |
| Amylase | - |
| Gelatinase | - |
| Urease | + |
| Cellulose | + |
| Protease | - |
| *Antibiotic susceptibility* |  |
| Penicillin | R (resistance) |
| Tetracycline | S (susceptible) |
| Streptomycin | S |
| Chloramphenicol | S |
| Nalidixic acid | S |

**Table S4:** Screening of bacterial isolates for production of plant growth promoting substances

| **Bacterial isolates** | **IAA production (µg mL^-1^)** | | | | | **ACC deaminase (μmol α-KB/mg Protein/h)** | **P-solubilization**  **(µg mL^-1^)** | **NH_3_ production** | **Siderophore (FeCl_3_ test)** |
| --- | --- | --- | --- | --- | --- | --- | --- | --- | --- |
|  | **0T** | **100T** | **200T** | **300T** | **400T** |  |  |  |  |
| KR-1 | 45.4 | 67.2 | 89.0 | 112 | 156 | 12.2 | 17.4 | ++ | ++ |
| KR-2 | 23.3 | 41.2 | 56.3 | 87.4 | 102 | 10.4 | 13.2 | + | ++ |
| KR-3 | 17.4 | 23.4 | 47.3 | 84.2 | 93.0 | 14.2 | 15.1 | + | ++ |
| KR-4 | 34.0 | 56.3 | 76.2 | 89.4 | 113 | 16.0 | 18.3 | + | ++ |
| KR-5 | 27.4 | 54.3 | 67.8 | 89.3 | 104 | 12.3 | 9.56 | ++ | ++ |
| KR-6 | 34.3 | 44.2 | 56.7 | 89.4 | 112.0 | 17.4 | 17.0 | ++ | ++ |
| KR-7 | 76.0 | 102 | 134 | 167 | 192 | 18.0 | 21.0 | ++ | ++ |
| KR-8 | 138 | 167 | 198 | 213 | 266 | 23.0 | 22.0 | ++ | ++ |
| KR-9 | 43.2 | 66.4 | 85.4 | 104 | 134 | 21.4 | 20.3 | ++ | ++ |
| KR-10 | 22.3 | 51.3 | 76.4 | 89.0 | 110 | 21.0 | 15.3 | ++ | ++ |
| KR-11 | 27.0 | 45.1 | 68.6 | 84.3 | 99.2 | 15.8 | 11.1 | + | ++ |
| KR-12 | 13.2 | 23.4 | 36.7 | 46.2 | 65.0 | 17.0 | 12.4 | + | ++ |
| KR-13 | 21.4 | 43.2 | 64.7 | 85.4 | 100.0 | 14.3 | 15.7 | ++ | ++ |
| KR-14 | 18.3 | 26.8 | 45.6 | 65.4 | 77.0 | 11.9 | 18.9 | + | ++ |
| KR-15 | 35.5 | 47.4 | 76.5 | 112 | 143 | 10.3 | 13.6 | + | ++ |
| KR-16 | 23.5 | 29 | 36 | 45 | 65 | 9.5 | 11.0 | + | ++ |
| KR-17 | 138 | 167 | 198 | 213 | 266 | 23.0 | 22.0 | ++ | ++ |
| KR-18 | 34.0 | 56.3 | 76.2 | 89.4 | 113 | 16.0 | 18.3 | + | ++ |

Each value is a mean (mean ±S. D) of three independent replicates

**Table S5-** Plant growth promoting active biomolecules synthesized by *K. radicincitans* under varying level of NaCl

| **Treatment** | **Dose rate (%)** | **Siderophore (% unit)** | **Zone of halo (mm)** | **Zone of solubilization**  **(mm)** | **Production of** | |
| --- | --- | --- | --- | --- | --- | --- |
|  |  |  |  |  | **HCN** | **NH_3_** |
| NaCl (w/v) |  |  |  |  |  |  |
|  | 0 | 23 ± 0.7 | 15 ± 0.6 | 18 ± 0.5 | **-** | **+++** |
|  | 2 | 26 ± 1.2 | 15 ± 1.3 | 17 ± 1.2 | - | **++** |
|  | 5 | 34 ± 2.1 | 16 ± 0.6 | 15 ± 0.6 | - | **++** |
|  | 7 | 39 ± 1.5 | 17 ± 0.5 | 14 ± 0.5 | - | **++** |
|  | 10 | 46 ± 1.8 | 17 ± 1.2 | 14 ± 2.3 | - | **+** |
|  | 12 | 53 ± 3.2 | 18 ± 0.6 | 13 ± 0.6 | - | ++ |
|  | 15 | 64 ± 2.5 | 20 ± 1.3 | 13 ± 0.6 | - | ++ |

Each value is the mean of three (n=3) ± standard deviation

**Supplementary Figure**

**Fig S1:** Neighbor-joined phylogenetic tree of *Kosakonia radicincitans* strain KR-17. The tree was constructed based on 16S rRNA partial gene sequence of selected PGPR and closely related phylogenetic species (type cultures) derived using NCBI BLAST search tool. Sequences were aligned using Clustal W sequence alignment tool in MEGA 7.0 software.

**Figure S2:** Biofilm development and its associated traits; biofilm formation (**panel A**), EPS production (**panel B**), swimming (**panel C**), swarming motility (**panel D**) alginate production (**panel E**) and cell surface hydrophobicity (**panel F**) of *K. radicincitans* KR-17 strain under varying levels of NaCl. In this figure, bar and line diagrams represents the mean values of three replicates (*n* =3). Corresponding error bars represents standard deviation (S.D) of three replicates (S.D, n = 3). The asterisks *, ** and # denote statistical significance at *p* < 0.05, *p* < 0.005 and *p* < 0.001, respectively computed by Student’s *t*-test.
